# Development and Genetic Monitoring of a Putatively Thiamine Deficiency Complex Tolerant Atlantic Salmon Broodstock

**DOI:** 10.1002/ece3.74122

**Published:** 2026-08-07

**Authors:** Kurt C. Heim, Avril M. Harder, Laurie A. Earley, Paul Boynton, Shane Hanlon, Jacques Rinchard, Kevin Kelsey, Stacey A. Nerkowski, Nathan R. Campbell, William R. Ardren

**Affiliations:** ^1^ U.S. Fish and Wildlife Service, Lake Champlain Fish and Wildlife Conservation Office Essex Junction Vermont USA; ^2^ College of Forestry, Wildlife and Environment Auburn University Auburn Alabama USA; ^3^ U.S. Fish and Wildlife Service White River National Fish Hatchery Bethel Vermont USA; ^4^ U.S. Fish and Wildlife Service Dwight D. Eisenhower National Fish Hatchery North Chittenden Vermont USA; ^5^ Department of Environmental Science and Ecology State University of New York Brockport Brockport New York USA; ^6^ Vermont Fish and Wildlife Department Ed Weed Fish Culture Station Grand Isle Vermont USA; ^7^ U.S. Fish and Wildlife Service Northeast Fishery Center Lamar Pennsylvania USA; ^8^ GTseek LLC Twin Falls Idaho USA; ^9^ U.S. Fish and Wildlife Service Silvio O. Conte National Fish and Wildlife Refuge Complex Hadley Massachusetts USA

**Keywords:** assisted evolution, Atlantic salmon, GT‐seq, parentage‐based tagging, thiamine deficiency complex

## Abstract

The concept of assisted evolution suggests that hurdles to species restoration in modified environments might be overcome by selective breeding of individuals better‐suited to the contemporary evolutionary landscape. Here, we introduce an experimental program aimed at developing an Atlantic salmon broodstock resilient to dietary thiamine deficiency complex (TDC). Dietary TDC is caused by overconsumption of lipid‐rich prey items containing thiaminase, leading to insufficient maternal allocation of thiamine to eggs and high offspring mortality. Within Lake Champlain, USA, where TDC is widespread due to consumption of invasive Alewife, we conducted offspring survival trials of 114 wild‐reared salmon pairs. We documented among‐family patterns in offspring survival to yolk‐sac absorption. Some females allocated sufficient free thiamine to eggs to avoid severe offspring mortality from TDC, while most did not and suffered complete or severe reproductive failure. Offspring from “resilient” families with high survival were used as founders of a putatively “TDC‐Tolerant” broodstock but at the expense of low effective population size (*N*_e_ = 48). We also developed a control broodstock that included additional families that only survived after supplemental thiamine treatment (MAX‐Gen, no selection, *N*_e_ = 152) to represent a status quo scenario. From 2016 to 2025 we (1) developed a Genotyping‐in‐Thousands by Sequencing (GT‐seq) marker panel (2) implemented parentage‐based tagging (3) curated two experimental broodstocks and released ~1.6 million of their offspring into Lake Champlain and (4) implemented a sampling program to monitor demographic and evolutionary outcomes. In Lake Champlain, TDC continues to be a major restoration bottleneck afflicting 42%–76% of salmon evaluated annually since 2007 and is an increasing conservation issue globally. Continuation of this work will determine whether assisted evolution improved TDC‐resistance in the wild and also evaluate the genetic and demographic consequences of contrasting broodstock strategies.

## Graphical abstract

We introduce efforts to develop a potentially thiamine deficiency complex (TDC) tolerant Atlantic salmon broodstock via selective breeding, as well as control broodstock with higher genetic diversity (and no selection). Additionally, we develop the genetic monitoring tools (novel genetic marker panel, parentage‐based tagging) to monitor the demographic and evolutionary outcomes of stocking offspring from these broodstocks (~1.6 million) in Lake Champlain, USA. Continuation of this work will determine if this approach improves TDC‐resistance in the wild.

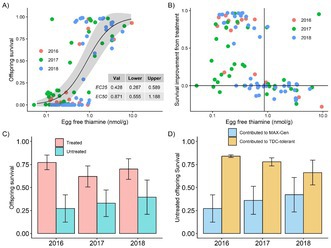

## 1 Introduction

Restoration of a population after local extirpation, through reintroduction, is difficult with success dependent on myriad factors (Kleiman 1989; Houde et al. 2015). First, addressing the root cause of extirpation is paramount (Cochran‐Biederman et al. 2015). If a population was extirpated owing to habitat loss, then habitat restoration is a prerequisite for reintroduction success. However, even if original causes of extirpation are managed, there are likely to be a suite of new challenges that have emerged since the species was extirpated. Invasive species, climate change, or changes in phenology of resources can all fundamentally alter the adaptive landscape to which the extirpated taxa were adapted (Visser and Both 2005; Hoffmann and Sgró 2011; Havel et al. 2015; Heim et al. 2019). The extent and nature of these changes need to be carefully considered when choosing a source population for restoration (Houde et al. 2015; Malone et al. 2018). Even if an exact genetic match to the extirpated population were available as a source, it might not do well: it was adapted to the historical adaptive landscape, which may be very different than the present‐day state of that same geographic location.

Given an appropriate source population is identified, one approach that may increase the rate of genetic adaptation to the contemporary evolutionary landscape is assisted evolution (Schlaepfer et al. 2005; Jones and Monaco 2009). Assisted evolution is broadly defined as intentionally enhancing adaptive traits to improve target species survival or reproductive success, often in modified environments, and typically involves some form of selective breeding (Schlaepfer et al. 2005; Jones and Monaco 2009). A prominent example focuses on improved heat tolerance to restore lost coral reefs or improve conservation of remaining ones (Van Oppen et al. 2015; Humanes et al. 2021). Application of assisted evolution in conservation would typically include (1) characterizing or identifying a specific bottleneck (e.g., increasing ocean temperatures) (2) using selective breeding or other tools to increase adaptive genetic variation relevant to this bottleneck and (3) developing a framework for monitoring success.

In this article we introduce an ongoing experimental program that seeks to use assisted evolution to reintroduce Atlantic salmon to Lake Champlain in the face of a substantial barrier to reproductive success, thiamine deficiency complex (TDC). Prior work has identified TDC as a major population bottleneck and uncovered potentially adaptive genetic variation underlying resilience to TDC (Ladago et al. 2020; Harder et al. 2020). Here we (1) use survival trials to identify TDC resilient individuals in the wild, (2) breed survivors together in a captive setting with the intention of increasing adaptive allele frequencies, and (3) release their offspring into the environment for monitoring. In addition, we report on the development of a suite of genetic monitoring tools to evaluate the success of this program.

### 1.1 The Bottleneck: Thiamine Deficiency Complex

Thiamine deficiency complex is emerging as a global conservation issue for salmonid populations (Ketola et al. 2000; Harder et al. 2018; Sutherland et al. 2018; Keinänen et al. 2018; Mantua et al. 2025). Thiamine (vitamin B1) is an essential coenzyme for many important functions (e.g., carbohydrate and fat metabolism, neurotransmitter synthesis, ATP production) that living organisms must acquire through their diet. A deficiency in thiamine results in a series of symptoms collectively referred to as TDC. In salmonids, symptoms are most severe for offspring of afflicted females, and include large yolk sacs with hemorrhaging, hydrocephalus, lethargy, irregular swimming behavior, and eventually death (Fisher et al. 1995; Fitzsimons et al. 2009). Thiamine deficiency complex has also been linked to poor migratory performance of adults and, in severe cases, can lead to direct adult mortality (Fitzsimons et al. 2005; Ketola et al. 2005; Harbicht et al. 2018). Reproductive failure of TDC afflicted salmonids has been implicated in demographic decline, and even complete extirpation, of populations across the globe (Karlsson et al. 1999; Ketola et al. 2000; Mantua et al. 2025).

Most cases of TDC in salmonid populations are associated with a diet including thiaminase‐rich prey items, lipid‐rich prey items, or both (Harder et al. 2018). Thiaminase is an enzyme that degrades thiamine in the gastrointestinal tract, thereby decreasing available thiamine for use in metabolism (Mitchell et al. 2021). Furthermore, consumption of lipid‐rich prey can lead to oxidative stress that reduces available thiamine in adults, as dietary free thiamine gets used up as an antioxidant (Lukienko et al. 2000; Keinänen et al. 2012). The clupeid fishes are a sort of double whammy—being both high in thiaminase activity and lipid‐rich—and are the most frequent taxon linked to TDC (Karlsson et al. 1999; Honeyfield et al. 2005). The increasing number of case studies documenting TDC, especially in already‐at‐risk salmonid populations, necessitates thoughtful management approaches to address this issue (Harder et al. 2024).

### 1.2 Contrasting Mitigation Approaches to TDC

Thiamine deficiency complex presents challenging issues for hatchery programs that collect wild‐reared adults to use as broodstock. If returning females used for broodstock have severe TDC then there would be (1) massive fry mortality, (2) excessive manual labor to remove mortalities, (3) difficulties meeting production targets, and (4) a severe loss of genetic diversity in the population. Fortunately, TDC can be mitigated via thiamine supplementation (Tillitt et al. 2005; Ladago et al. 2020). Thiamine injections of females, or immersion of eggs or fry in a thiamine bath, are highly effective at increasing offspring survival (Fitzsimons et al. 2005; Harbicht et al. 2018; Ladago et al. 2020). Some hatchery programs have responded to TDC by integrating a thiamine treatment into annual broodstock and egg take operations. However, widespread treatment relaxes selection for potential TDC resilience. If there is adaptive genetic variation related to TDC tolerance, then it could conceivably be driven to higher frequency in the wild (via evolutionary rescue) or by hatchery managers using selective breeding (Kosch et al. 2022; Harder et al. 2024). However, there are important genetic risks to consider, as selective breeding will invariably reduce genetic diversity within the population.

Harder et al. (2024) concluded that the appropriate management approach to TDC is context dependent. In most cases, the goal should be to support maximum genetic diversity through thiamine supplementation. Treatments ensure as many spawning broodstock contribute offspring to the next generation and lowers variance in family size contributions, increasing *N*_e_. Harder et al. (2024) suggest that moving forward with the “genetic adaptation” approach (i.e., omit thiamine supplementation, with hopes that this encourages adaptation to TDC resistance) should be reserved for scenarios where there is evidence of inheritable TDC tolerance and the consequences of reduced genetic diversity are acceptable. Atlantic Salmon in Lake Champlain fit both these criteria and represents an excellent system to test the genetic adaptation hypothesis.

### 1.3 Atlantic Salmon in Lake Champlain

Recent evidence from landlocked Atlantic salmon in Lake Champlain suggests there may be a genetic basis for TDC tolerance (Harder et al. 2020, 2024). Harder et al. (2020) conducted survival trials of offspring of TDC‐afflicted salmon and identified notable among‐family variation in offspring survival. Moreover, genomic analysis revealed 1446 putatively adaptive genes that were associated with offspring survival, some with a mechanistic link to a probable role in TDC tolerance (e.g., neurogenesis and visual perception). These results provide a strong basis for exploring assisted evolution and the unique historical context of the Lake Champlain salmon population make this an ideal scenario for experimentation. This population was extirpated in the early 1800s and thus the distinct evolutionary lineage is already lost. Furthermore, ongoing restoration of self‐sustaining populations efforts are severely limited by a widespread TDC that is attributable to the introduction of Alewife ( *Alosa pseudoharengus* ) in 2003, which have become the predominant prey item for Atlantic salmon and Lake Trout ( *Salvelinus namaycush* ) (Simonin et al. 2018; Ladago et al. 2020). Additionally, emerging techniques like parentage‐based tagging (PBT, Steele et al. 2019) and cost‐efficient genotyping methods such as Genotyping‐in‐Thousands by sequencing (GT‐seq, Campbell et al. 2015) provide viable means to evaluate the success of this approach on a large‐scale in a natural environment.

### 1.4 Study Objectives

The goal of this article is to introduce ongoing efforts to develop and monitor a salmon broodstock that is more tolerant to TDC in Lake Champlain. We report on this collaboration among multiple agencies (2016–present) in several sections:

#### 1.4.1 Section 1: Family Level Survival Trials

We conducted survival trials of wild‐reared fish offspring to examine among‐family variation in TDC resistance. Our objectives were to (1) identify maternal factors related to offspring survival, (2) develop updated egg‐thiamine thresholds required for 50% and 25% offspring survival (i.e., EC_50_ and EC_25_), (3) identify TDC‐associated single nucleotide polymorphisms (SNPs) for inclusion in a novel genetic marker panel and, (4) select “survivors” to use as founders for a TDC‐Tolerant broodstock.

#### 1.4.2 Section 2: Curation of Experimental Broodstocks and Implementation of PBT

We describe the development of an experimental TDC‐Tolerant broodstock (low *N*_e_) and a control broodstock without selection for TDC tolerance (MAX‐Gen, high *N*_e_). In short, we identified “survivors” (Section 1.4.1) and bred this limited set of individuals together, intentionally inducing a genetic bottleneck in hopes of increasing adaptive allele frequencies. The control broodstock (MAX‐Gen) incorporated both the “survivors” as well as individuals who only survived when administered supplemental thiamine as eggs. We also describe the process used to implement PBT and monitor the performance of these fish in the wild (Steele et al. 2019).

#### 1.4.3 Section 3: Development of GT‐Seq Panel, Validation, Genetic Variation in Broodstock

We report on a novel GT‐seq marker panel that was developed to perform genetic parentage assignments (for PBT), track TDC‐associated SNPs, and identify genetic sex. In this section we describe (1) how SNPs were selected for inclusion in the panel, (2) the panel's performance in parentage and genetic sex assignments, and (3) provide a preliminary assessment of allele frequency differences attributable to our selective breeding program. We compare allele frequencies of both broodstock lineages with the “full” gene pool of broodstock founders as an initial assessment of how selection and drift contribute to genetic change.

#### 1.4.4 Section 4: Thiamine Monitoring

We report on long‐term thiamine monitoring of Atlantic salmon in Lake Champlain to evaluate temporal patterns in TDC severity and incidence rate, comparing values to updated EC_50_ and EC_25_ estimates derived in Section 1.4.1.

## 2 Methods

***Study Area***

Following glacial retreat and the draining of the Champlain Sea (~12,000 years ago), formerly anadromous Atlantic salmon evolved a landlocked life history within Lake Champlain, migrating up rivers to breed and attaining adult size in the forage‐rich lake environment (Hutchings et al. 2019). However, due to dam construction, habitat loss, and overharvest, Lake Champlain salmon were extirpated by the early 1800s (Marsden and Langdon 2012). Reintroduction programs began in the 1970s, using fish sourced from a variety of other extant landlocked salmon sources but predominantly Sebago Lake, Maine (Harder and Christie 2022). The stated goals of these stocking programs are to maintain a sport fishery and maximize the contribution of wild fish (Hurst et al. 2020). Stocking has created a popular sport fishery, but wild recruitment was absent or severely limited for the first ~45 years of this program.

The introduction of Alewife in 2003 led to a widespread shift in Atlantic salmon diet and emergence of severe TDC that was first documented at the Ed Weed Fish Culture Station (Grand Isle, Vermont), a facility run by the Vermont Department of Fish and Wildlife (VTDFW, Simonin et al. 2018; Ladago et al. 2020). Here, smolt‐stocked fish rear in Lake Champlain and return as adults to the hatchery (i.e., feral fish), where they are collected and used for broodstock. Thiamine treatment of all eggs collected from feral fish was initiated in 2007 to alleviate massive offspring die‐offs associated with TDC. Despite TDC and limited evidence of wild recruitment resulting from stocking efforts for over 45 years (e.g., 1970–2016), in 2016 and 2017 surviving wild fry were first sampled in the Boquet and Winooski Rivers (Prévost et al. 2020). This represented the first confirmed wild reproduction in over 150 years and was regarded as a major milestone for restoration efforts. It was also surprising given the severity of TDC in the Lake (Ladago et al. 2020). Concurrent with this discovery of wild fry, Harder et al. (2020) revealed among‐family variation in TDC tolerance and a probable genetic basis for TDC tolerance, and TDC had become a more globally relevant conservation issue (Harder et al. 2018; Sutherland et al. 2018; Mantua et al. 2025). Thus, the U.S. Fish and Wildlife Service (USFWS) initiated development of an experimental TDC‐Tolerant broodstock.

### 2.1 Family Level Survival Trials

In summary, this section describes how we selected TDC‐resilient individuals to use as founding individuals for an experimental broodstock lineage. We obtained gametes from stocked salmon that reared naturally in Lake Champlain and returned to Ed Weed Fish Culture Station. Gametes were transported to White River National Fish Hatchery (Bethel, VT) where milt and eggs were combined to form a total of 128 crosses for offspring survival monitoring (*n* pairs: 2016 = 17, 2017 = 51, 2018 = 60, Table 1). Importantly, the parents used for gamete collection were reared to adult size naturally within Lake Champlain, consuming natural and potentially TDC‐inducing prey items. The 128 families include all of those reported in Harder et al. (2020, *n* = 35) plus an additional 93 families not previously reported on. A sample of eggs from all females in 2016 and 2018, and 35 (out of 60) in 2017, was collected prior to fertilization and frozen and stored at −80°C until shipment to either USGS Northern Appalachian Research laboratory or SUNY Brockport for thiamine analysis. Concentrations of three thiamine vitamers (TH, free thiamine; TPP, thiamine pyrophosphate; TMP, thiamine monophosphate) were quantified following methods described in Futia et al. (2017). Additionally, we calculated total thiamine (THH) as the sum of TH, TMP, and TPP for use in subsequent analysis.

**TABLE 1:** Captive broodstock spawning history at White River National Fish Hatchery.

BY	Source	Spawning note	Gen	Contributing families	
				MAX‐Gen	TDC‐tolerant
2016	Feral	Survival trials	F1	17	5
2017	Feral	Survival trials	F1	44	16
2018	Feral	Survival trials	F1	44	28
2019	Captive	16F × 17M	F2	50	46
2020	Captive	16F × 17M & 17F × 16M	F2	50 (25, 25)	50 (25, 25)
2021	Captive	17F × 17M & 18F × 18M	F2	222 (47, 175)	219 (54, 165)
2022	Captive	18F × 18M & 19F × 19M	F2/F3	191 (61, 130)	189 (60, 129)
2023	Captive	19F × 19M & 20F × 20M	F3	260 (60, 200)	259 (44, 215)
2024	Captive	20F × 20M & 21F × 21M	F3	250 (50, 200)	250 (50, 200)

*Note:* In the “spawning note” column we describe the origin of the individuals spawned in that brood year (BY). For example, in 2016–2018 spawners were sourced from feral fish included in survival trials. In 2019, the offspring of BY16 (females) were spawned with offspring of BY17 (males). The “Gen” column indicates number of generations in captivity and the contributing families column shows the total number of spawners per BY, followed by the number of different BY combinations corresponding to “spawning note.”

For biosecurity purposes we treated all fertilized eggs with a 0.005% iodophor solution for 30 min and then rinsed them with fresh water to prepare for treatment. We divided fertilized eggs from each family into two groups, soaking one group in a 1% thiamine mononitrate solution for 30 min (hereafter, “treated”) and the other in water for 30 min (“untreated”), and transferred them to individual heath trays for survival observation. Total eggs per family used in survival evaluation varied annually and across families due to availability from Ed Weed Fish Culture Station (mean ± SD: 2016 = 1534 ± 812; 2017 = 381 ± 213; 2018 = 764 ± 171). At ~50 days postfertilization, we began weekly tracking and removal of mortalities until ~130 days postfertilization and after yolk‐sac absorption. Individuals alive at this final checkpoint were considered to be surviving offspring. In further analysis of survival, all eggs that did not survive to yolk‐sac absorption were counted as mortalities (e.g., inviable eggs removed on Day ~50, as well as those removed thereafter) to be consistent with methods in Harder et al. (2020). In 2018, an entire egg‐rearing unit had to be eliminated due to fungus growth (14 families), leaving a total of 114 families total that are considered further for survival estimates.

We used time‐to‐event survival analysis to estimate the survival of each family and treatment, and to develop an index of family level risk of death associated with TDC. Data were first converted to an individual time‐to‐event format for each egg, where each egg is an individual (*n* = 90,990) for which an event (either [1] death or [2] censorship) was recorded in association with an event time in units of days‐since‐fertilization. Individuals were censored if they were removed from heath trays alive (e.g., RNA sequencing, Harder et al. 2020). The “survfit” function from the R package “survival” (Therneau 2015) was used to estimate survival for each family and treatment group, and log‐rank test (α = 0.05) was used to determine if treatment with a thiamine bath significantly affected survival within each family (Kleinbaum and Klein 2012; R Development Core Team 2026). After calculation of survival estimates for each family, we compared these to the associated egg thiamine concentration of that family using Spearman's nonparametric rank correlation (i.e., Spearman's rho, ρ).

We calculated the relative family level risk of death of untreated individuals using a Cox proportional hazards regression implemented with the “coxph” function of the “survival” package (Therneau 2015). The estimated hazard ratio represents the relative risk of death associated with belonging to a particular family, with the baseline risk of 97.3% (i.e., 2016‐family‐1, as in Harder et al. 2020). We used linear regression to test for the effect of maternal thiamine concentration, maternal weight, and year on family level risk of death. We evaluated six models, each including both maternal weight and year (2016, 2017, 2018) as fixed effects, and one metric of maternal thiamine, either TH, THH, or TH/THH (i.e., ratio of free thiamine to total thiamine) as non‐transformed or log‐transformed values as fixed effects. We used AIC to select the top model for interpretation of effects. We also calculated the effective concentration required for 25% and 50% survival (EC_25_, EC_25_) from a fitted dose–response curve estimated as described in Harder et al. (2020) using the “drc” package in R (Ritz et al. 2015). Here we estimated dose–response curves with (1) THH (for consistency with Harder et al. 2020) and (2) with the thiamine vitamer with the strongest correlation to survival identified in our results.

### 2.2 Curation of Experimental Broodstocks and Implementing PBT

Following completion of survival trials (2016–2018), aliquots of surviving fry from untreated families were counted, combined, and reared to sexual maturity in circular raceways at White River National Fish Hatchery. This now‐captive lineage is henceforth called the “TDC‐Tolerant” broodstock. Individuals in the TDC‐Tolerant broodstock were first spawned in 2019 when the females resulting from the 2016 spawn (now age 3) were mated with 2017 males (now age two, Table 1). Cross year class spawning was performed to avoid potential mating of full siblings in the 2016‐year class, which would be expected to occur by chance in 1/5 pairings for the TDC‐Tolerant lineages (i.e., it consisted of only 5 founding families). During each year of spawning (2019—present), offspring produced are used jointly for stocking fry and smolts in Lake Champlain and to continue the broodstock lineage in a captive setting. To continue the lineage, an equal number of eyed eggs from each family are combined and reared at the hatchery. We also collected aliquots of surviving fry from as many families as possible (from the same survival trials in 2016–2018) to develop a control broodstock (MAX‐Gen). Here, families that only survived with supplemental treatment were also included. Importantly, the MAX‐Gen broodstock thus included all families present within TDC‐Tolerant (i.e., the survivors), but with the addition of those families that only survived owing to thiamine treatment. The MAX‐Gen and TDC‐Tolerant lineages have remained fully separated while in captivity. The TDC‐Tolerant and MAX‐Gen broodstocks generally represent the two alternative management responses outlined in Harder et al. (2024). The full spawning history (2016—present) including the number of spawning pairs annually is depicted in Table 1.

We provide descriptive statistics to contrast the two broodstock lineages and calculated the effective number of breeders (*N*_b_) annually for each (2016–2018). With the founding parent information (i.e., mating pairs, mean number of eggs per family contributing to broodstocks [$\bar{K}$], and sex ratios), we used the demographic estimates of *N*_b_ adjusted for variance in family size (σ*K*) and unequal sex‐ratios presented in (Ardren and Kapuscinski 2003). We also calculated the single‐generation *N*_e_ for the two broodstocks using the methods of Waples (2002) which accounts for differential reproductive success by each of the three brood years that founded each broodstock (2016–2018). Estimated *N*_e_ was also expressed as the ratio of *N*_e_ to the census size of spawning adults (*N*_c_, *N*_e_/*N*_c_). In this section we also provide a description of how PBT was implemented.

### 2.3 Development of GT‐Seq Panel, Validation, Genetic Variation in Broodstock

#### 2.3.1 SNP Selection

We developed a genotyping‐in‐thousands by sequencing (GT‐seq) marker panel for the primary purpose of genetic parentage analysis to facilitate PBT (Campbell et al. 2015). Secondary intended purposes were to determine genetic sex, provide a set of markers associated with TDC tolerance, and examine markers associated with sea‐age (Ayllon et al. 2015). We cover these details in File S1 and provide a general description of the process here. First, we collected restriction‐site associated DNA sequencing (RAD‐seq) data from 95 of the founding individuals used in our two broodstocks—these included fish that did (*n* = 53) and did not (*n* = 42) produce surviving offspring in the absence of a thiamine treatment. We used SNPs identified from the RAD‐seq data (*n* = 78,410 with minor allele frequency > 0.05) and binary offspring survival data (i.e., any offspring survived = TRUE/FALSE) to perform association tests to identify SNPs associated with offspring survival for (1) just females and (2) both sexes. We developed a list of SNPs ranked based on the strength of their association with offspring survival. We then identified SNPs (from within this ranked list) that were (1) in or near differentially expressed genes identified in Harder et al. (2020), (2) resulted in a nonsynonymous change in an annotated gene, or (3) located in an intron of an annotated gene or resulted in a synonymous change in an exon (i.e., potential regulatory consequences). In sum we selected 146 putatively TDC‐associated SNPs, ~500 polymorphic SNPs (with high minor allele frequencies, to use for parentage assignments), and 21 SNPs associated with sea‐age (from published literature) for potential inclusion in a GT‐seq maker panel that was designed by GTSEEK (https://gtseek.com).

#### 2.3.2 Parentage Performance and Genetic Sex ID

To validate the effectiveness of our panel for genetic parentage assignment and sex identification, we genotyped a set of 2444 samples at the completed GT‐seq panel. Sequencing was done on a NextSeq 1000 (Illumina Inc.) and 8.3% of samples (one column from each 96‐well plate of extracted DNA) were additionally run on a MiSeq (Illumina Inc.) as a means to determine laboratory error rate (File S1). Samples included (1) all 95 ascertainment samples (i.e., that RAD‐seq was performed on, whose offspring participated in survival trials, and were founders of our captive broodstocks), (2) all hatchery broodstock from MAX‐Gen and TDC‐Tolerant lineages that were spawned in 2019, 2020, and 2021 at White River National Fish Hatchery (*n* = 1930 broodstock samples, Table 3), and (3) 419 PBT validation samples from genetically tagged fish immediately prior to stocking (2021–2023). We began releasing genetically tagged fish from MAX‐Gen and TDC‐Tolerant lineages, in equal proportions, into Lake Champlain tributaries in 2021 (Table S3). Prior to each release we implemented a standard sampling protocol where genetic samples are collected from 5 to 10 individuals per rearing unit. Here, these fish represent individuals with parents that are also genotyped (i.e., hatchery broodstock) and provide a useful dataset to test the accuracy of our panel for parentage assignments. Additionally, annual collection, genotyping, and PBT assignment of these validation samples serve as a means to assess PBT tagging rates and detect unintentional mixing of release groups.

As a first examination of the panel for genetic parentage assignments, we used 1930 broodstock genotypes, 419 offspring off these broodstock (i.e., validation samples), and 32 negative (“control”) genotypes that were known to not be parents of the offspring. The 32 control genotypes were from the 2018 feral adults collected at Ed Weed Fish Culture Station used as broodstock founders. We next identified parent‐offspring trios using the likelihood‐based approach implemented in the program SNPPIT (Version 1.0; Anderson 2012). Initial runs using the full suite of available loci suggested non‐Mendelian patterns of inheritance in some markers, thus we iteratively eliminated some SNPs from analysis to achieve a final set of 176. After identifying a final set of 176 SNPs, we excluded genotypes with > 10% missing data and performed a final assignment run. Using assignments made by SNPPIT, we allowed a single mendelian incompatibility per parent‐offspring trio to allow for genotyping error. If no likely trio was identified, or if the most likely trio exhibited > 1 mendelian incompatibility, parentage was not assigned. We then compared assigned parents from this analysis to our database of spawning broodstock pairs, and identified valid assignments (i.e., assigned pair matched a TRUE pair known to have spawned), and invalid assignments. To determine the accuracy of our genetic sex markers, we compared genetic sex to phenotypic sex using all fish used as broodstock which were of known sex.

#### 2.3.3 Genetic Variation in Broodstocks

One of our stated aims in developing the TDC‐Tolerant broodstock was to increase potentially adaptive alleles relative to their initial frequency in the population. Our final GT‐seq marker panel included 35 putatively TDC‐associated SNPs discovered via RAD‐seq and association tests using survival data (see above). We refer to these as “putatively” TDC‐associated SNPs because their association with offspring survival in the context of TDC has not yet be verified in independent samples. Here we use these SNPs to perform a simple test to determine how allele frequencies have changed in our TDC‐Tolerant broodstock, and our control broodstock (MAX‐GEN), relative to the full parental gene pool of founders (i.e., feral fish used in survival trials 2016–2018). We performed Chi‐squared tests (α = 0.05) for each loci, calculated mean change in minor allele frequency, and used *t*‐tests to evaluate differences in allele frequency change magnitude between groups. In chi‐squared tests, we do not make corrections for multiple hypothesis testing (e.g., Bonferroni correction) but rather report the expected number of Type I errors expected (based on the number of tests performed) and compare this to the number of observed significant tests (Waples 2015). Lastly, we also used a discriminant analysis of principal components (DAPC) to visualize differences among groups using the dapc() function from “adegenet.” While the putatively TDC‐associated SNPs allele frequencies are expected to change in the TDC‐Tolerant broodstock (relative to the founding gene pool owing to selection) – genetic drift will also play a role in genetic divergence. Although we do not attempt to statistically distinguish the role of selection and drift in these exploratory analyses, we perform the same sequence of allele frequency comparisons described above using the remaining polymorphic loci in our SNP panel (i.e., those used for parentage, and presumably neutral). Additional allele frequency comparisons between (1) founders that did and did not produce surviving offspring and (2) MAX‐Gen and TDC‐Tolerant groups are provided in the Supporting Information.

### 2.4 Thiamine Monitoring

Egg thiamine concentration monitoring of wild fish began in 2004 to track TDC severity and trends over time (Ladago et al. 2020), and we present an update to this time series in this article. Unfertilized eggs from ~30 females were collected annually from Atlantic salmon returning to Ed Weed Fish Culture Station in early to mid‐November and analyzed for thiamine concentration as described above. We used a Kruskal–Wallis test followed by Dunn's post hoc test to identify significant differences between 2004 samples, before alewife proliferation, and samples from subsequent years. We also compare the values to EC_50_ and EC_25_ developed from survival trials to determine the incidence of TDC at different rates across years. We used EC_50_ and EC_25_ values based on TH and THH. We also explored variation in different thiamine vitamers and ratios of vitamers visually.

## 3 Results

### 3.1 Family Level Survival Trials

**FIGURE 1:**
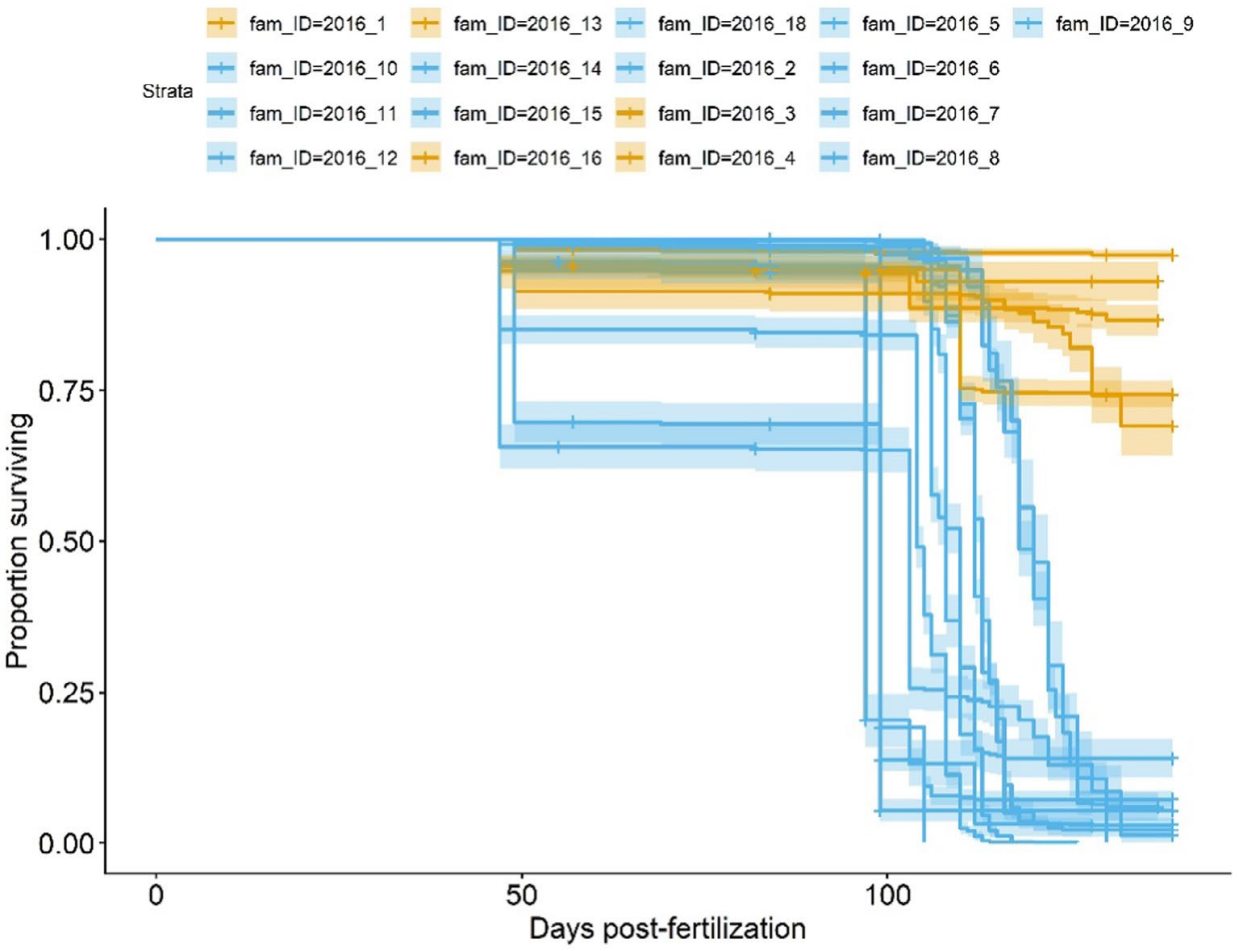
Offspring survival of feral landlocked Atlantic salmon adults collected from Lake Champlain in 2016 showing symptoms of thiamine deficiency complex (TDC). Each line represents cumulative survival of a unique family of fertilized eggs through yolk sack absorption (family IDs listed as strata). Yellow lines indicate families that contributed to the TDC‐Tolerant broodstock (high survival, resilient to TDC), blue lines represent families that did not contribute to the TDC‐Tolerant broodstock (low survival).

**FIGURE 2:**
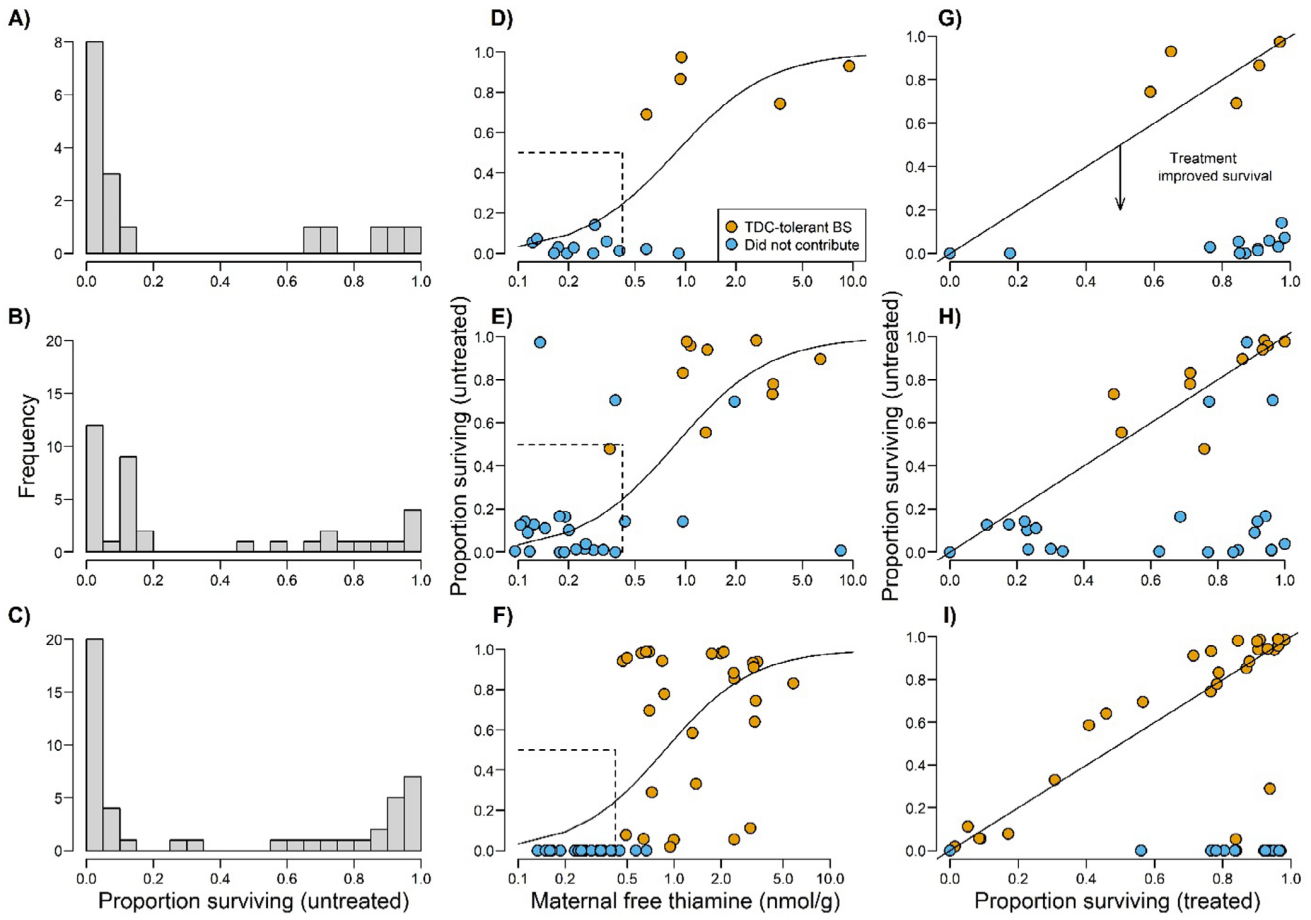
Distribution of family level survival to ~130 days post fertilization in 2016 (panel A), 2017 (panel B), and 2018 (panel C) for feral fish families not treated with supplemental thiamine. The second column of plots shows survival in relation to maternal contribution of free thiamine to eggs (panel D = 2016, panel E = 2017, panel F = 2018) with dashed lines indicating EC_50_ and a fitted dose response curve. The third column of plots shows offspring survival for each family with and without supplemental thiamine treatment (panel G = 2106, panel H = 2017, panel I = 2018). The 1:1 line across the plot is shown for reference; points below this line indicate that thiamine treatment improved survival. Yellow colored dots are families that contributed to the TDC‐Tolerant broodstock, blue dots are families that did not contribute to the TDC‐Tolerant broodstock.

Survival of families without supplemental thiamine treatment was strongly bimodal (Figures 1 and 2A–C). Families survived very well (25% of families had survival > 80%), or poorly (60% of families had survival < 20%), and only a small proportion had intermediate values (16% of families had 20%–80% survival, Figure 2A–C). Additionally, 29% of families produced no surviving offspring at all (33/114) (Figure 2A–C).

**FIGURE 3:**
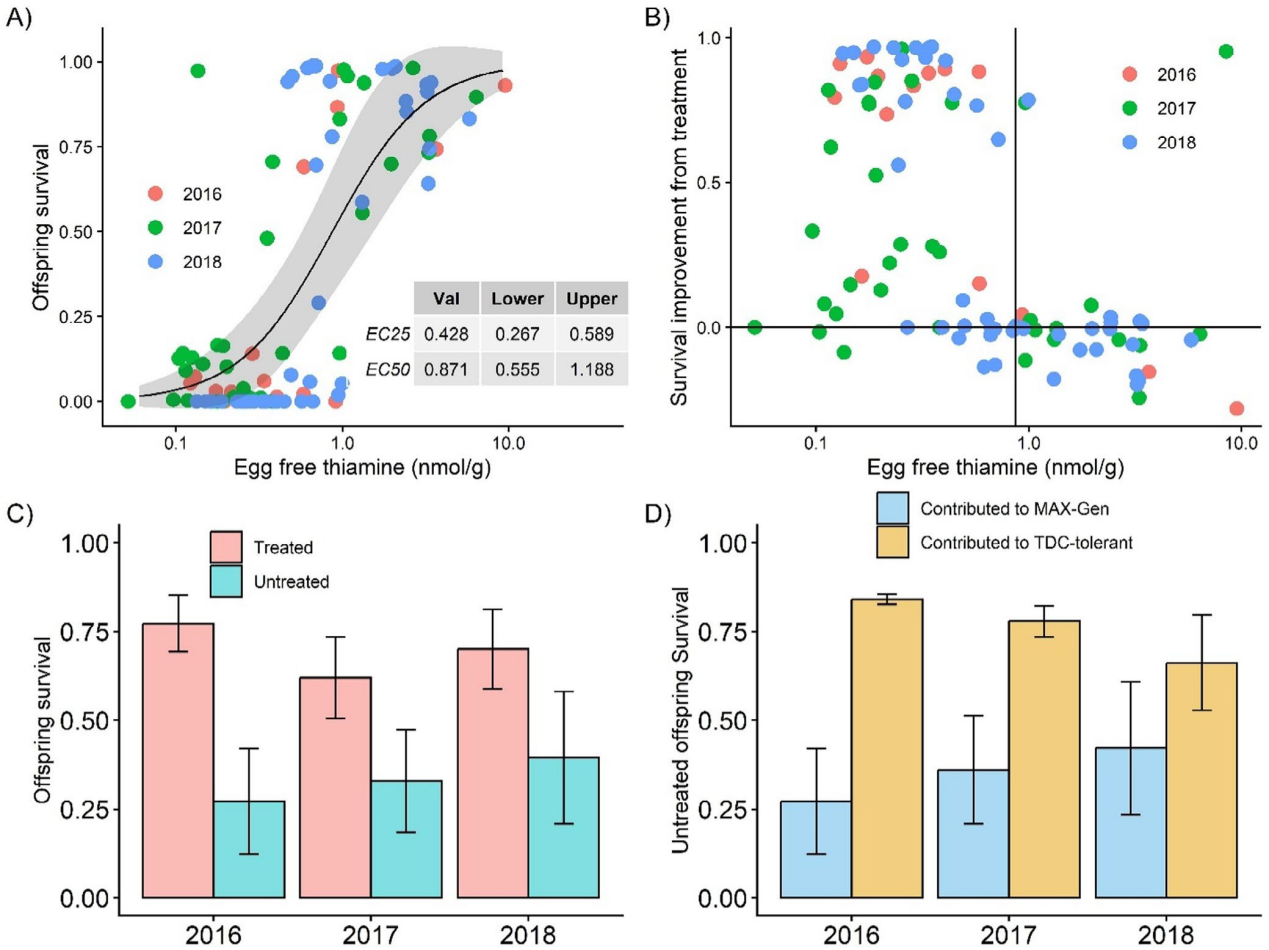
Aggregate dose response curve from family‐level survival data collected in 2016–2017 (panel A) and survival improvement from thiamine treatment in relation to starting maternal thiamine contribution (panel B). Panels C depict overall offspring survival (with variance) of treated and untreated families, and panel D shows mean and variance in survival for individuals that contributed as founders of the MAX‐Gen and TDC‐Tolerant broodstocks.

Offspring survival was strongly associated with egg TH (Spearman's rank correlation, ρ = 0.59, *p* < 0.001, Figure 2D–F, Figure S1). Total thiamine (e.g., THH = TH + TMP + TPP) was also significantly associated with offspring survival (ρ = 0.32, *p* < 0.001), as was TMP (ρ = 0.26, *p* = 0.007). Thiamine pyrophosphate was not significantly associated with offspring survival (ρ = 0.09, *p* = 0.37). The ratio of TH/THH and TH/TPP were strong indicators of offspring survival (TH/THH: ρ = 0.63, *p* < 0.001; TH/TPP: ρ = 0.61, *p* < 0.001). The top dose–response model (two‐parameter log‐logistic function) suggested an EC_25_ and EC_50_ of 0.42 nmol/g (95% CI: 0.26–0.59) and 0.87 nmol/g (0.55–1.18) using TH (Figure 3A). This means that a TH of at least 0.43 nmol/g is the minimum predicted concentration required for 25% of a female's offspring to survive and 0.87 nmol/g is required for 50% offspring survival. Using the THH (for comparison to Harder et al. 2020), estimated values were 2.98 nmol/g (2.04–3.95) for EC_25_ and 5.47 nmol/g (3.92–7.02) for EC_50_ (also using a two‐parameter log‐logistic function).

The top model (of six tested) of family‐level risk of death, based on AIC, was one that included a log‐transformation of TH (ΔAIC next best model = 4.09). This model outperformed one including TH without a log‐transformation by 20.04 AIC units, strongly suggesting a nonlinear effect of TH on risk of death. This model highlighted that the only significant predictor of family level risk was TH, whereas year and maternal weight had no effect (Table 2, Figure S2).

**TABLE 2:** Multiple regression on family level hazard ratios (i.e., risk of death from 0 to ~130 days post fertilization).

Term	Estimate	SE	*T*	*p*
Intercept	4.11	0.71	5.81	< 0.001
Year‐2017	−0.52	0.63	−0.82	0.410
Year‐2018	0.68	0.56	1.12	0.23
Log(TH)	−1.19	0.19	−6.32	< 0.001
Maternal weight	−0.44	0.38	−1.15	0.23

*Note:* The year 2016 is the baseline for covariate year which was treated as a factor.

Treatment with a 30‐min thiamine supplement generally increased offspring survival, but most notably in families with initially low maternal thiamine (Figure 3B). Embryo survival was significantly higher in treated eggs (compared to untreated eggs from the same family, log‐rank tests) in 76% of families in 2016 (13/17), 50% of families in 2017 (25/50), and 40% of families in 2018 (19/47). Families with maternal thiamine below EC_50_ for TH experienced significantly higher survival improvements from thiamine treatment (median survival improvement = 76%) than those families with maternal thiamine above EC_50_ (median = 0%, *t* = 6.6, *p* < 0.001, Figure 3B).

### 3.2 Curation of Experimental Broodstocks and Implementing PBT

**FIGURE 4:**
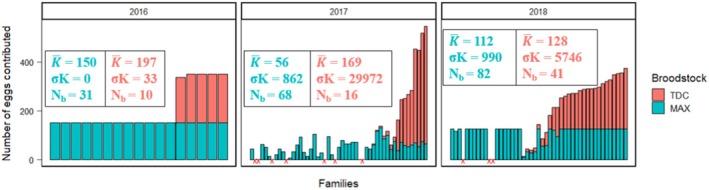
Family level contribution of eggs (from feral adult parents) that contributed to two experimental broodstocks (TDC‐Tolerant and MAX‐Gen) in 2016–2018. Red Xs indicate families that were evaluated but contributed to neither broodstock due to complete offspring mortality even with supplemental treatment. Within each panel inset box we show mean eggs contributed per family ($\bar{K}$), variance in egg contributions per family (σ*K*), and the effective number of breeders (*N*_b_).

Following completion of survival trials, aliquots of eggs from untreated families were counted, combined, and reared to adult spawning size; these are henceforth called the TDC‐Tolerant broodstock (Figure 4; Table 3). Over the 3 years (2016–2018), this included contributions from 49 families (98 individuals) with high family‐level survival without treatment ($\bar{S}$ = 0.72) and low among‐family variance in survival (σ*S* = 0.09). The overall Ne of the TDC‐Tolerant BS was 48.02 which is roughly half of the census size (*N*_c_) of spawning adults (*N*_e_/*N*_c_ = 0.49). A notable reduction in annual *N*_b_ occurred in 2017 due to high variation in family level egg contributions ($\bar{K}$= 169, σ*K* = 29,972, Figure 4). Although 32 families contributed offspring and the sex‐ratio was equal, high σ*K* reduced annual *N*_b_ to 16 (Table 3). The MAX‐gen broodstock was founded by more families (105 families, 205 individuals) and had lower and more variable family‐level survival for untreated offspring ($\bar{S}$ = 0.37, σ*S* = 0.17). This low mean and high variance occurred because many families had offspring survival of 0% without treatment, but were still allowed to contribute to MAX‐Gen because some of their eggs were also treated (and thus, survived). The overall *N*_e_ of the MAX‐gen broodstock was 151.87 (*N*_e_/*N*_c_ = 0.74). Annual *N*_b_ of MAX‐Gen was 3.1 (2016), 4.25 (2017), and 2.0 (2018) times higher than the TDC‐Tolerant broodstock. Untreated family‐level survival of contributing families was 3.1 (2016), 2.1 (2017), and 1.57 (2018) times higher for TDC‐Tolerant broodstock relative to MAX‐gen (Table 3).

**TABLE 3:** Summary of founders of two experimental broodstocks (2016–2018) named TDC‐tolerant and MAX‐Gen.

Year	*N*‐tot	THH	TH	% below EC_50_ (TH)	TDC‐tolerant				MAX‐Gen			
					$\bar{S}$	σ*S*	*N*	*N*_b_	$\bar{S}$	σ*S*	*N*	*N*_b_
2016	17	3.83 (2.65)	1.14 (2.31)	70%	0.84	0.01	10	10.03	0.27	0.15	31	30.90
2017	51	2.96 (2.45)	1.07 (1.83)	66%	0.78	0.04	32	15.66	0.36	0.15	88	67.85
2018	47	5.82 (1.87)	1.17 (1.26)	62%	0.66	0.13	56	41.85	0.42	0.19	88	82.21

*Note:* The columns are as follows: % below EC_50_TH, the percent of females annually that had TH below EC_50_; $\bar{S}$, mean survival of untreated offspring to ~130 days post fertilization; *N* = total number of individuals, and *N*_b_ the effective number of breeders; *N*‐tot, total number of families included in annual survival trials; TH, free thiamine concentration per female followed by standard deviation; TTH, average total thiamine concentration per female (nmol/g) (followed by standard deviation); σ*S*, variance in offspring survival.

Surviving fry from survival trials (i.e., the first generation born in captivity, F_1_s) were reared at White River National Fish Hatchery to spawning age and first used for egg collections in brood year (BY) 2019. The broodstock lineages have continued in captivity with the BY‐2024 spawn representing the third generation in captivity (F_3_s, Table 1). Annual egg collections are jointly used for (1) production of fish for stocking in Lake Champlain and (2) for use as future broodstock. Starting in BY 2020, PBT had been fully implemented to distinctly mark 18 unique release groups for stocking and monitoring in Lake Champlain (Table S3). At spawning (White River National Fish Hatchery), genetic samples are collected from each single parent broodstock cross and distinct families of embryos are tracked through eye‐up (Figure S3). Lots of approximately 25–35 known and unique contributing families are combined to form a “PBT‐group” (Figure S3) that is either sent to Dwight D. Eisenhower National Fish Hatchery to be reared to 1.5‐year‐old smolts (smolt release) or sent to Adirondack Fish Hatchery (New York Department of Environmental Conservation) to continue incubation for a spring release as unfed fry in NY tributaries to Lake Champlain. In this manner, we annually create 18 distinctly marked release groups (9 MAX‐Gen and 9 TDC‐Tolerant) for a series of concurrent applied studies of stocked fish in Lake Champlain (see Section 4).

### 3.3 Development of GT‐Seq Panel, Validation, Genetic Variation in Broodstock

#### 3.3.1 SNP Selection

The final GT‐seq panel (i.e., after SNP selection by GTSeek) included 211 markers (164 polymorphic loci for parentage, 35 SNPs putatively associated with TDC tolerance, nine SNPs derived from published literature, and three markers targeting the *S. salar* sex‐determining gene (*sdY*) (Figure S4)). We reviewed data for the first genotyping run of 2444 fish to describe the marker panel characteristics. We did not observe genotype differences between our initial run and our QA/QC run. Among 164 polymorphic SNPS, three were not variable and one failed to produce any genotypes. Considering the remaining 160 markers, 10.6% of markers had more than 10% missing data and mean minor allele frequency of the variable markers was 0.32. Among the putatively TDC‐associated SNPs, 34 were variable and one failed to produce any genotypes. Results related to the sea‐age SNPs generally suggest little to no variation at these markers in our population (Table S1). Preliminary parentage analysis (considering all polymorphic loci, excluding *sdY* markers) led to a selection of a final 178 markers for use in parentage after removal of SNPs with high rates of mendelian incompatibilities.

#### 3.3.2 Parentage Performance and Genetic Sex ID

For parentage analysis validation we first screened data to require a 90% genotyping rate, where 2415/2444 (98.8%) met this criterion at the selected set of 178 loci to be used for parentage. The average genotyping completion rate per sample at these markers was 99.5% (i.e., 177/178 loci genotyped), with individuals missing between 0 (*n* = 1203) and 18 (*n* = 1) loci. Samples retained included 1982 broodstock samples, 401 validation samples, and 32 “control” samples whose parents were known to not be in the dataset. Using SNPPIT for parentage assignment, 394/401 of the validation samples (98.3%) were assigned to true parent pairs (i.e., male—female IDs that matched a pair known to have been crossed during spawning), 6/401 (1.5%) failed to assign to parents, and one (0.2%) was assigned to an invalid parent pair (i.e., male—female IDs known to NOT have been crossed during spawning). None of the 32 negative control samples assigned to parents. Additionally, all assigned pairs were matched to the expected PBT group from which they were collected (394/394), which indicates that we detected no unintentional mixing of PBT groups during the hatchery rearing process.

The three *sdY* sex markers were 100% consistent with one another, and 100% consistent with phenotypic sex recorded during spawning out of 1930 genotyped broodstock. Missing data at these markers were low, with two samples failing in SJI_SEX (0.1%), four failing in SdyE2.2_SEX (0.2%), and two failing in Sdy_E3_SEX (0.01%).

#### 3.3.3 Genetic Variation in Broodstock Lineages

**FIGURE 5:**
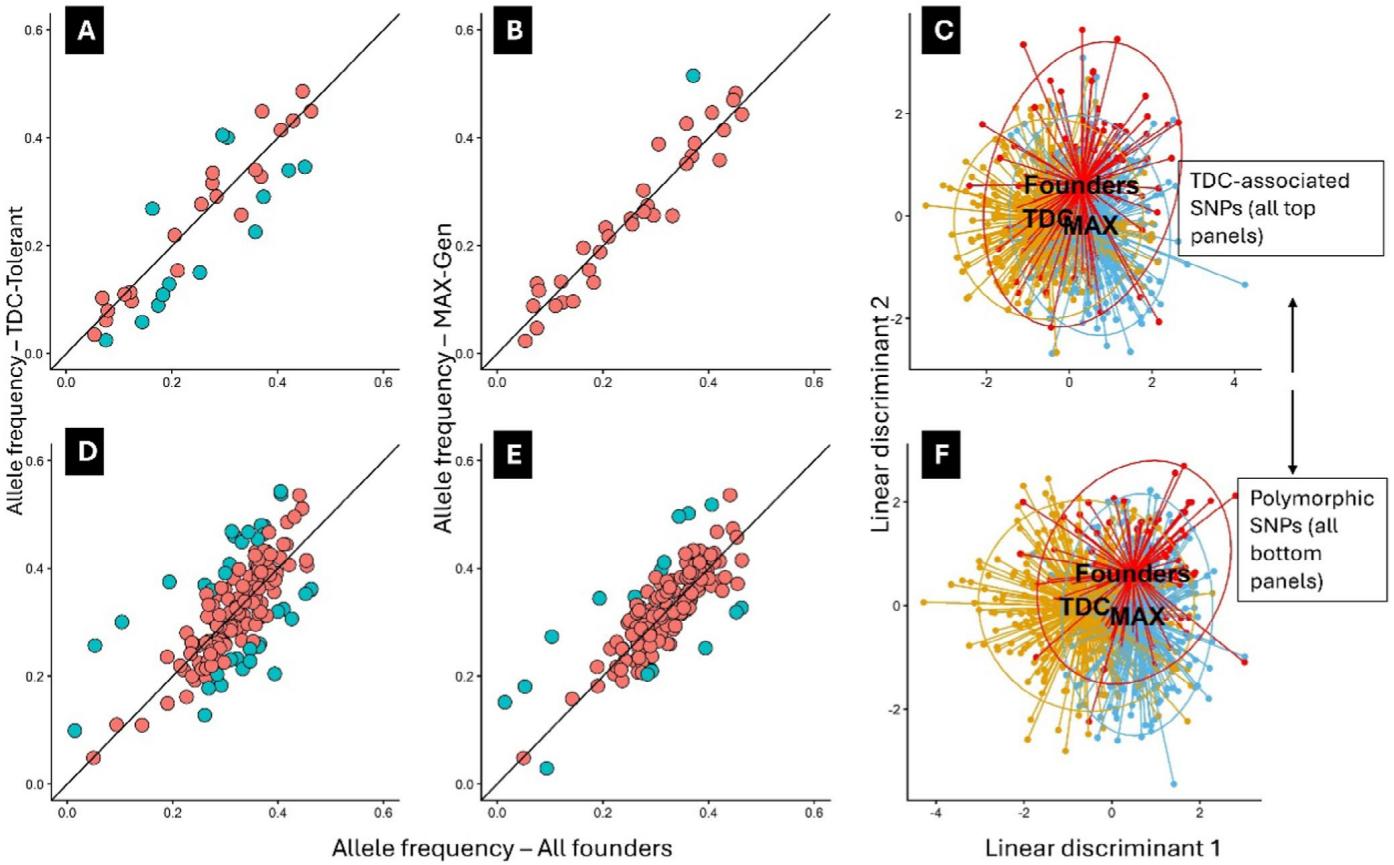
Allele frequency differences between all broodstock founders and resulting broodstock lineages (MAX‐Gen, TDC‐Tolerant). Each point in panel A, B, D, E is an individual loci with top panels depicting results for TDC‐associated SNPs (*n* = 34 shown) and bottom panels show data for polymorphic loci used for parentage (*n* = 163 shown); within a panel, loci that differed significantly are shown in blue, while nonsignificant loci tests are shown in red. Panels C and F show visualizations from discriminant analysis of principal components using the TDC‐associated SNPs (C) or polymorphic SNPs (F).

Considering the 35 TDC‐associated SNPs, allele frequencies differed significantly (*p* < 0.05) at 41% (13/34) loci when comparing the TDC‐Tolerant lineage (*n* = 329 individuals, offspring of BY 2019–2021) to the pre‐mating gene pool of broodstock founders (*n* = 95, Figure 5A). In contrast, only 3% (1/34) of these SNPs differed significantly in the MAX‐Gen lineage (*n* = 339, Figure 5B) in relation to the parent gene pool. In the two aforementioned tests, roughly two significant tests would be expected by chance when testing 34 loci. In addition, the mean allele frequency difference was greater between TDC‐Tolerant lineage and founders (mean = 0.051, SD = 0.038) than it was when comparing MAX‐Gen to founders (mean = 0.032, SD = 0.28, *T* = −2.25, *p* = 0.027). The DAPC visualization showed some separation, but groups did not show discrete clusters.

At the polymorphic loci used for parentage, allele frequencies differed significantly at 23% of loci (37/159) when comparing the TDC‐Tolerant lineage (same sample sizes as above) to the pre‐mating founder gene pool. The same comparison with MAX‐Gen showed 11% of loci were significantly different (18/159); in these comparisons roughly eight tests would be expected to be significant owing to chance alone. The mean difference between TDC‐Tolerant and founders in allele frequencies (mean = 0.054, SD = 0.043) was greater than the differences between MAX‐Gen and founder allele frequencies (mean = 0.040, SD = 0.04, *T* = −3.06, *p* = 0.002). The DAPC visualization using these SNPs showed slightly more separation than the one using TDC‐associated SNPs; however, there remained substantial group overlap.

### 3.4 Thiamine Monitoring

**FIGURE 6:**
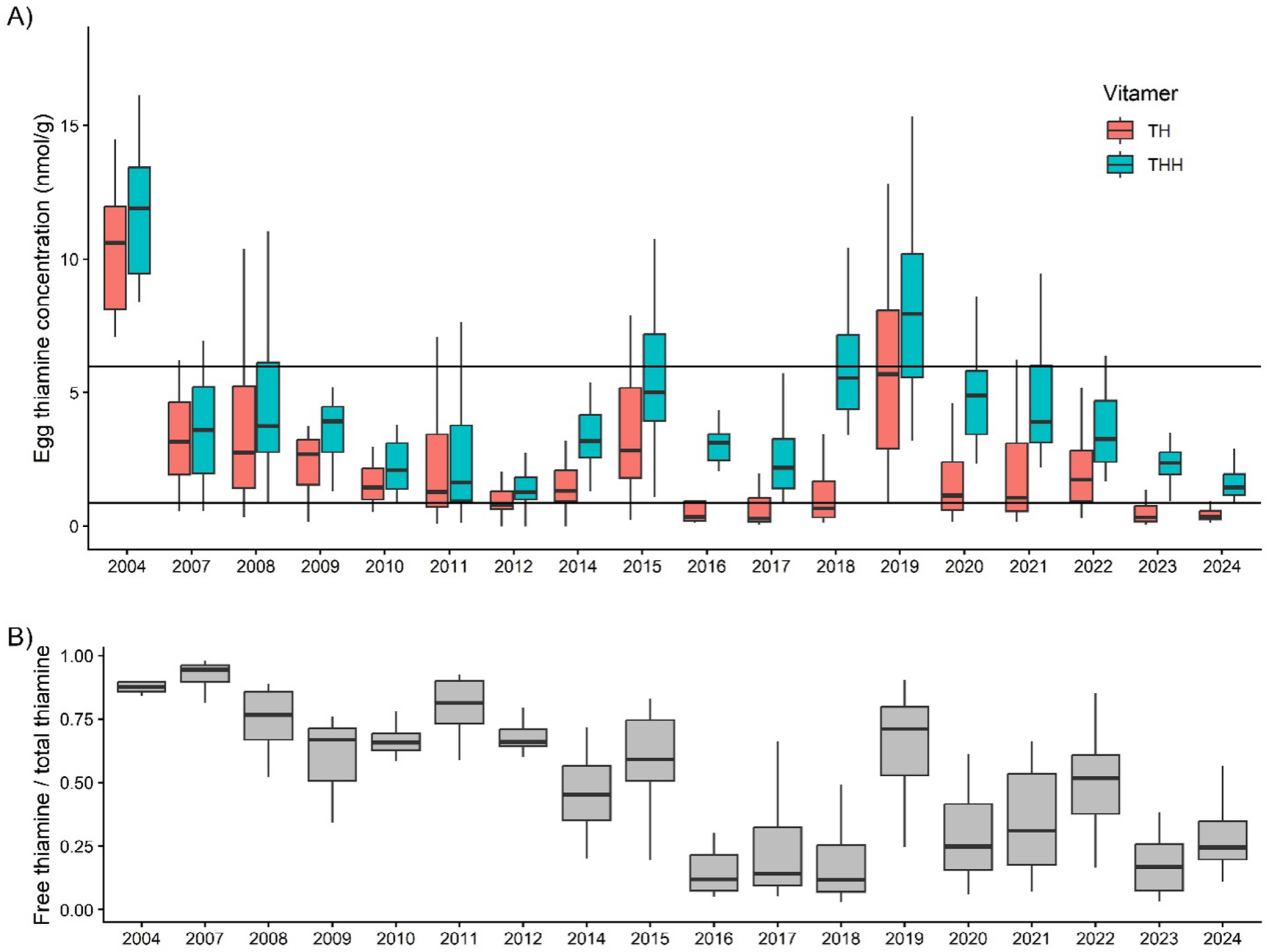
Trends in free thiamine (TH) and total thiamine (THH) concentration of landlocked Atlantic salmon eggs collected from feral returns to Ed Weed Fish Culture Station (panel A). Lines at EC_50_ (total thiamine) and EC_50_ (free thiamine) are shown in panel A at 0.87 nmol/g and 5.47 nmol/g, respectively. Panel B shows the ratio of TH to THH.

Prior to widespread Alewife proliferation in Lake Champlain (2004), median THH was 11.90 nmol/g, and 8% of samples (1/12) were below EC_50_(THH) (Figure 6A). By 2007 Alewife had proliferated and the median THH decreased to 3.59 nmol/g, and 81% were below EC_50_(THH) (21/26). Egg thiamine values post‐alewife invasion (2007–2024) compared to 2004 levels were lower in 94% (17/18) of statistical comparisons (Dunn's post hoc tests), only approaching the 2004 level in 2019 where median THH was 7.92 nmol/g and not statistically different than values in 2004 (11.90). Considering all post‐2004 samples, 75.5% of samples tested were below EC_50_(THH) and 40% were below EC_25_(THH). Additionally, in all but 2 years (2018, 2019), more than half of tested fish were below EC_50_(THH) (Figure 6A).

The trends in TH over time were not entirely concordant with THH trends, owing to inconsistent TH/THH ratios over time (Figure 6B). Accordingly, estimates of TDC incidence based on TH were lower than those derived from THH. Median TH in 2004 was 10.60 nmol/g and 0% of samples were below EC50. By 2007, TH had decreased to 3.15 nmol/g and 15% (4/26) were below EC_50_(TH). Egg TH values post‐alewife invasion (2007–2024) compared to 2004 levels were lower in 94% (17/18) of statistical comparisons, only approaching the 2004 level in 2019 (median = 5.68 nmol/g). Considering all post‐2004 samples, 42% of samples tested were below EC_50_(TH) and 24% were below EC_25_(TH). In 6 years (2012, 2016–2018, and 2023–2024) at least half of all individuals tested, within that year, were below EC_50_(TH).

## 4 Discussion

We expand on the adaptive potential strategy for source selection outlined in Houde et al. (2015) by using assisted evolution modeled after the concept of evolutionary rescue (Carlson et al. 2014; Van Oppen et al. 2015). In the face of a population bottleneck, this approach includes survival trials of test subjects within the target environment to identify those with high fitness and then using captive breeding to bring potentially adaptive genetic variation to a higher frequency. We implement this approach in the context of widespread TDC, which is a growing conservation concern globally for salmonids and other taxa (Sutherland et al. 2018; Mantua et al. 2025). In our survival trials, we demonstrated bimodal patterns in family‐level offspring survival where maternal TH explained 59% of the observed variation. The mechanisms leading to differential thiamine allocation and offspring survival among families remain unknown; however, the important point is that we identified and used resilient individuals for founders of an experimental broodstock. Moreover, use of this survival data and paired RAD‐seq SNP discovery allowed us to identify putatively TDC‐associated SNPs for further testing. The remainder of our article describes the development and validation of genetic and demographic monitoring tools to monitor the success of this program moving forward.

### 4.1 Context of Lake Champlain Salmon Management and Genetic Goals

The initial motivation for this work was a recognition that TDC‐induced mortality was a major bottleneck to Atlantic salmon restoration in Lake Champlain (Harder et al. 2020; Ladago et al. 2020). Although not an initial factor contributing to extirpation, it remains a key challenge even as progress is made on other issues. For example, access to spawning habitat is increasing via dam removals (Hill et al. 2019), fish passage is being addressed at regulated dams (Nyqvist et al. 2017), and invasive Sea Lamprey ( *Petromyzon marinus* ) control is improving (Young et al. 2021). However, because Alewife now dominates Atlantic salmon diets (Simonin et al. 2018), even if other stressors are alleviated, achieving self‐sustaining populations may still be elusive using a status quo broodstock program.

We highlight the already‐extirpated status of Lake Champlain salmon early in this discussion to contrast our approach to TDC‐mitigation with restoration and supplementation programs elsewhere. The goal of most hatcheries working with threatened and endangered species is to prevent extinction by enhancing natural reproduction with a focus on preserving genetic diversity (Fisch et al. 2015). Attaining these goals is complex owing to the inevitable genetic consequences of captive rearing (Araki et al. 2007; Blanchet et al. 2008; Fraser 2008), but many populations would be lost if not for these programs. Our scenario is different as Atlantic salmon in Lake Champlain are not considered threatened or endangered, and management instead focuses on restoration of ecosystem services which include ecological function, recreational fishing, and harvest (in addition to promoting natural reproduction). From a hatchery management perspective, this different context gives us opportunities to explore approaches that would be inappropriate elsewhere. The risks of failure (e.g., failure to improve TDC‐resistance) are acceptable and without permanent consequence to an extant and unique evolutionary lineage. Additionally, what we learn here may inform TDC‐mitigation strategies elsewhere, where consequences of different approaches have more lasting and irreversible evolutionary and demographic consequences (Harder et al. 2024; Mantua et al. 2025).

### 4.2 Among Family Variation in Offspring Survival

We documented among‐family variation in offspring survival trials of wild‐reared Atlantic salmon, generally corroborating Harder et al. (2020); however, this larger dataset revealed (1) a notable bimodal distribution in offspring survival and (2) a stronger link between TDC and maternal thiamine. This bimodality is consistent with a maternally transmitted disease because 59% of variation in offspring survival was explained by maternal TH. Moreover, those families with the worst survival and lowest egg thiamine values saw the greatest benefits of supplemental thiamine treatment. The effectiveness of TDC treatments is well established (Werner et al. 2006; Ladago et al. 2020), and in our study it verified that much of the mortality we observed was attributable to TDC.

Maternal TH was a better predictor of offspring survival than other thiamine vitamers (TMP, TPP) or THH. This result differs from Harder et al. (2020) who found no association between offspring survival and maternal traits (including THH) in nine families also analyzed for RNA expression. This difference is likely explained by the inclusion of more families in our analysis (114 vs. 9), and because only THH was tested by Harder et al. (2020). While we did find a relationship with THH, it was weak. Other studies with Atlantic salmon and other species of salmonids also point to TH as the best predictor of TDC‐induced offspring mortality (Keinänen et al. 2018; Futia and Rinchard 2019; Vuorinen et al. 2021). Therefore, we conclude that using TH for monitoring incidence rate of TDC in Lake Champlain is more favorable than using THH.

Using TH, we estimated an EC_50_(TH) of 0.87 nmol/g (0.55–1.12) which is higher than that reported for Baltic Sea salmon (0.47 nmol/g, 0.45–0.49), which may be related to differing levels of exposure (i.e., potential selection for TDC resistance). In Baltic salmon, TDC was first identified in 1974, and thus exposure to TDC has been ongoing for quite some time (Karlsson et al. 1999), potentially allowing a longer period for selection for TDC‐resistance. There is some, albeit limited, previous work identifying population‐level adaptations to TDC‐resilience supporting this hypothesis (Mitchell et al. 2021; Fitzsimons et al. 2021). In Lake Trout, EC_50_ values may be lower in some stocks with a longer history of reliance on Alewife (Fitzsimons et al. 2021). This study also found more efficient utilization of TH within developing eggs (i.e., slower rate of use) in some populations. This implies that given a low starting concentration of TH (i.e., maternal allocation), some populations may be able to survive longer (i.e., efficiency) until exogenous feeding can provide dietary thiamine. A similar study with Atlantic salmon found no evidence for among population variation in offspring survival in three populations fed an experimental diet to induce TDC (Mitchell et al. 2021). However, a limitation in Mitchell et al. (2021) is that the experimental diet was only able to reduce THH slightly (11 nmol/g) which is not sufficiently low to induce widespread TDC and may have prevented detection of population‐level differences in TDC‐resilience. Our new estimates of EC_50_(TH) will be highly useful in monitoring incidence of TDC moving forward in Lake Champlain; additionally, the estimates presented here will serve as a useful benchmark. If adaptation towards more efficient thiamine utilization does occur in the future, a decrease in EC_50_ values could be an important indicator.

Alternatively, differences in EC_50_ estimates among populations may be attributable to how survival was estimated. For consistency with Harder et al. (2020), a variable number of inviable eggs per family were included as mortalities. The alternative is to consider these as background mortality and only estimate TDC‐associated survival using a starting sample of eyed eggs. We believe the overall consequences of this choice are minimal in our study as inviable egg removals were limited—for example, in 2016 these removals can be seen as rapid decreases in cumulative survival on Day 50 (Figure 1). Families that survived well (or didn't) would have quite similar outcomes; however, this raises the important point that the details of how survival is estimated are thoroughly described.

So why are some families within the same population, within the same year, apparently resistant to TDC? First, it may be that some salmon do not eat as many Alewives as others due to difference in prey selection or spatial variation in habitat use and co‐occurrence with Alewives. Links between TDC and foraging locations have been discovered elsewhere, supporting this hypothesis (Karlsson et al. 1999; Keinänen et al. 2018). Variation in maternal egg thiamine (and offspring survival) may also be related to diet variability of adult salmon (i.e., what else, besides Alewives are being eaten?), because thiaminase mediated thiamine degradation is dependent on other cofactors (Edwards et al. 2023). Certain cofactors (e.g., vitamin B3) can severely inhibit the ability of thiaminase to degrade thiamin, and if these can be acquired by certain individuals it might explain observed difference in egg thiamine concentrations even if Alewife remain the predominant prey item in Lake Champlain (Simonin et al. 2018). Aside from behavioral mechanisms (i.e., prey selection and encounter rates), some studies also indicate that TDC‐inducing properties of prey (e.g., high thiaminase and lipid content) vary across space and time. For example, thiaminase activity of Alewife in Lake Michigan varied across locations, seasons, and Alewife size (Tillitt et al. 2005) and Fitzsimons et al. (2005) found widely ranging thiaminase activity in 10 surveyed freshwater stocks. Studies from the Baltic Sea region suggest that variation in prey lipid content (the hypothesized causative factor of TDC in the region) across time and space is predictive of TDC related fry mortality and egg thiamine concentration (Keinänen et al. 2012). In summary, variation in offspring survival (2016–2018) and egg thiamine concentration (2007–2024) is likely related to differences in individual diet and prey properties (thiaminase activity, lipid content) that vary across space and time. Additionally, non‐dietary factors, including individual variation in thiamine allocation among females, thiamine utilization rates, or other genetic factors unrelated to maternal thiamine allocation may have contributed to observed variation (Honeyfield et al. 2005; Harder et al. 2020; Edwards et al. 2023). Although we do not yet understand what drives this variation, the important points relative to our broodstock program are that some individuals appear resistant to TDC and we identified these families and used them as broodstock founders.

### 4.3 Development of Two Experimental Broodstocks

We developed two captive broodstock lineages that generally mimic two contrasting mitigation approaches to TDC in salmonids (1) maintain high *N*_e_ (i.e., manage for maximum survival) and (2) allow for adaptation (i.e., manage in a way to facilitate TDC‐resistance) (Harder et al. 2024). To our knowledge, this is the first program attempting to test the adaptation hypothesis. By only using survivors as founders for the TDC‐Tolerant broodstock, we sacrificed genetic diversity at for the potential benefit of an immediate fitness benefit. Estimated *N*_e_ of this broodstock was 49, and is below commonly recommended thresholds (e.g., 50 or 100) suggested to avoid immediate fitness losses (e.g., 5 generations) from inbreeding (Frankham et al. 2014). The actual *N*_e_ is likely much smaller: though we were able (in some years) to equalize egg contributions per family, differences in family level egg survival in captivity will undoubtedly occur (Allendorf 1993). Although having roughly 1/3 the *N*_e_ of our control broodstock (MAX‐Gen, *N*_e_ = 149), the TDC‐Tolerant broodstock was founded by parents with offspring survival 1.5–3.1 times higher than MAX‐Gen in the context of 3 years with notably high rates of TDC. Hence, by selecting only these “survivors” with potentially adaptive genetic variation for continued breeding, we aimed to increase adaptive allele frequency (i.e., via survivor × survivor matings within the TDC‐Tolerant lineage) and facilitate evolutionary rescue by releasing their offspring back into the wild. A potential limitation of our strategy is that each cohort of founders (i.e., 2016, 2017, 2018 feral fish used in survival trials) was only subject to a single, albeit strong, selection event before entering a captive setting with relaxed selection for TDC‐tolerance. We do not currently have the capacity to maintain a feral return program supporting annual survival challenges of untreated offspring to induce multiple generations of selection; however, such a program could be explored should our approach fail to produce appreciable differences in TDC‐resiliency between broodstocks.

### 4.4 Novel GT‐Seq Marker Panel Performance

We developed a new GT‐seq panel to use for genetic parentage analysis, tracking putatively TDC‐associated SNPs, genetic sex identification, and evaluating patterns in sea‐age associated genes in our population. Overall performance for parentage assignments of the panel was excellent in preliminary testing using a subset of 178 SNPs, which is expected given that 100–200 SNPs are typically adequate for assignment of parent‐offspring trios (Flanagan and Jones 2019). Incorporating genotyping failure rate, as well as failed assignments, we achieved an overall parentage‐based tagging rate of 98.3% which is similar to or better than rates reported elsewhere (Steele et al. 2019). We also highlight that the genetic sex markers were 100% concordant with phenotypic sex determined from broodstock samples, consistent with recently developed sex markers elsewhere (e.g., 97% in Robertson et al. 2024). Combined with an excellent record of hatchery organization (e.g., we detected no unintentional mixing of hatchery release groups), this provides an excellent tool for continued monitoring of these experimental broodstocks and release groups into the future (Table S3).

### 4.5 Preliminary Assessment of Allele Frequency Differences

One of our underlying goals was to increase potentially adaptive allele frequencies in the TDC‐Tolerant broodstock to release “resilient” fish back into Lake Champlain, and our results (although preliminary) do suggest we have (1) identified some TDC‐associated SNPs as well as (2) increased their frequency in the TDC‐Tolerant broodstock relative to their availability in the founder gene pool (and, their frequency in the MAX‐Gen lineage). First, it is entirely expected that the TDC‐associated SNPs would be differentiated between founders that did and did not produce surviving offspring (e.g., Figure S6 and File S1: RESULTS). This is because these same individuals with paired survival data were used to discover these SNPs via RAD‐seq in the first place, and, significant associations between SNPs and survival could have arisen by chance. To confirm an association to TDC resilience, it is necessary to perform survival trials with new individuals and determine if these SNPs can predict maternal TH or offspring survival. Assuming that these SNPs are associated with TDC‐tolerance, we were successful in bringing them to a higher frequency via our family‐level selection trials and breeding program (e.g., 41% of loci were different relative to founders). Moreover, we were successful in maintaining the “status quo” allele frequencies in MAX‐Gen as only 1 of these loci differed significantly from the founder gene pool. While these putatively TDC associated loci demonstrate the effects of family level selection, significant allele frequency differences at the remaining polymorphic loci (i.e., those selected for parentage analysis) highlight the effects of genetic drift. As expected, given the lower *N*_e_ of TDC‐Tolerant broodstock, drift appears to have played a larger role in genetic differentiation relative to MAX‐Gen. More loci were significantly different, and, the magnitude of the shift in allele frequencies was significantly greater.

### 4.6 Thiamine Trends in Lake Champlain

Our time series monitoring of egg thiamine status highlights within and among year variations in TDC incidence rate, but also that the perceived severity depends on whether EC_50_ (TH) or EC_50_ (THH) is used. Temporal trends in median THH suggest a cyclical pattern with an immediate decline following Alewives proliferation (2007–2012), a rise‐fall pattern from 2012 to 2017, and another rise‐fall pattern from 2016–present. We note, however, that despite this temporal fluctuation, 75% of all tested samples were below EC_50_ (THH) and in 15/17 years more than half of fish tested would be expected to show signs of TDC. The perspective provided by TH and comparisons to EC_50_ (TH) are also concerning, but generally less so than the perspective provided by THH. Based on TH, 42% of sampled fish are below EC_50_ (TH) and in 6/17 years median TH was below EC_50_ (TH). The inconsistency between these two metrics across years is in part owing to temporal variation in TH/THH ratios: TH and THH are not entirely synchronous in their trends. In other words, temporal trends in the three vitamers making up total thiamine (TH, TMP, TPP) are not consistent (Figure S5). During the early part of the time series (2007–2013) trends in THH and TH were generally synchronous, because TH made up the majority of THH which is typical in salmonids without severe TDC (Honeyfield et al. 2005; Futia and Rinchard 2019). However, in recent years TH/THH ratios have fluctuated, decoupling trends in TH and THH. Other studies have noted that the ratio of TH to THH (and/or other specific vitamers) is often lower in individuals that have TDC (Wolgamood et al. 2005; Keinänen et al. 2018, 2022). Although beyond the scope of this study, further exploration of factors influencing ratios of vitamers and their relative role in TDC are warranted. Regardless, because TH has the strongest relationship with offspring survival in our study, we plan to use TH to examine incidence rates of TDC moving forward.

In all, continued thiamine monitoring emphasizes that TDC remains a critical challenge to restoration, within and among year variation in egg TH is common, and that fortunately, our survival trials were conducted in 3 years where selection pressures for TDC resilience were expected to be strongest. In 2016–2018, when survival trials were conducted, 64% of fish were below EC50 (TH) and 47% were below EC25 (TH).

### 4.7 Future Directions and Opportunities

By curating these broodstocks, implementing PBT to identify distinct release groups, annually releasing approximately 357,000 individuals (147,000 smolts, 210,000 fry, Table S3), and conducting widespread sampling within Lake Champlain, we set up a large‐scale experiment to address the following topics in subsequent research.

How well do MAX‐Gen and TDC‐Tolerant broodstock lineages perform following release in terms of survival, growth, and return return‐rates to rivers? Although the expected advantage of the TDC‐Tolerant broodstock is increased reproductive success (not, necessarily their own survival), there is potential that other life history traits may be influenced. First, it is possible that selection for TDC‐Tolerance has also, inadvertently, selected for other traits that may be either beneficial or detrimental in the wild. Second, the lower *N*_e_ of the TDC‐Tolerant broodstock could more rapidly lead to inbreeding depression that could negatively impact post‐release survival or other important traits. Average inbreeding (F) within the TDC‐Tolerant broodstock is expected to accumulate three times faster than MAX‐Gen and at a rate of ~0.01 per generation (assuming *N*_e_ = 49, and Fisher‐Wright idealized population assumptions, ∆*F* = 1/2*N*_e_, Wang et al. 2002). In reality, it will likely accumulate faster than this and could reach levels of 0.05–0.10 in a few generations where detrimental effects on salmonid populations are known to occur (Wang et al. 2002). The design of our lake‐wide experiment and use of PBT will allow us to monitor inbreeding and its effects on performance‐related traits in both a hatchery and wild setting. Using PBT, we can assign recaptured individuals to both a PBT group (e.g., which broodstock, what age at stocking, what stocking location) as well as a family with genotyped parents. We can then test if broodstock membership or parental inbreeding coefficients have an effect on measurable performance traits. Moreover, continued performance monitoring will provide important information to determine when introduction of new genetic material in the captive lineage may be necessary (e.g., outbreeding, or a genetic “refresh”).

A fundamentally important question to answer is whether the TDC‐Tolerant broodstock has a higher relative reproductive success than MAX‐Gen. Differences in reproductive success between broodstocks can be estimated in several ways and at different life stages: First, in a controlled setting at White River National Fish hatchery, we have completed data collection for an experimental feed trial to induce TDC in MAX‐Gen and TDC‐Tolerant individuals for an offspring survival comparison (2021–2024). Using a low‐thiamine diet including amprolium (a thiamine antagonist) (Fynn‐Aikins et al. 1998) administered to all fish, this study focuses on identifying differences in survival, egg thiamine allocation, and EC_50_ between broodstocks that are unrelated to differences in dietary choice or foraging behavior. Second, within Lake Champlain, we have established recapture programs to collect genetic samples from adult returns to three rivers (Winooski River, Boquet River, Saranac River), mark redds, and sample surviving offspring the following year. With these data, we can use multigenerational PBT or grandparentage analysis to estimate relative reproductive success between MAX‐Gen and TDC‐Tolerant broodstocks. Initially we plan to estimate relative reproductive success by assigning fry and parr to parents; however, our program is currently set up in a way to potentially estimate relative lifetime reproductive success. It is exciting to report that during our spring sampling in 2025, we sampled over 200 putatively wild salmon that can be used for this analysis.

Our PBT release groups also include nested studies to examine the relative contribution of fry vs. smolts, the effects of a “soft‐release” relative to a hard release, and the effects of stocking location (e.g., upstream vs. downstream) on adult return rate and straying (Heim et al. 2025, Table S3). Each of these nested studies uses PBT as a marking framework and was developed by collaboration with fisheries managers in the region. Moreover, each nested study is providing data to answer the questions noted above (i.e., survival and reproductive success of contrasting broodstocks) because both TDC‐Tolerant and MAX‐Gen fish are being released in equal proportions across stocking locations (Table S3). We hope that these studies can guide future stocking decisions in Lake Champlain and elsewhere where Atlantic salmon restoration efforts are ongoing.

### 4.8 Assisted Evolution and Hatchery Management

There is widespread evidence that hatchery programs have led to rapid genetic changes for spawning timing, age at maturity, growth rates, and behavioral traits that most often have negative effects on remaining wild fish (Tipping and Busack 2004; Araki et al. 2007, 2008; Saikkonen et al. 2011). Recognition of such harmful genetic change has led to a paradigm shift in hatchery management: do everything to limit genetic change imposed by breeding practices (Allendorf 1993; Fisch et al. 2015; Bartron et al. 2018). Here, we recognize the capacity of hatcheries to influence evolution and are attempting to use them in a targeted manner to overcome a population bottleneck. In practice, similar programs exist; for example, those that curate disease‐tolerant trout broodstocks for reintroduction programs (Hedrick et al. 2003; Baerwald et al. 2011), or artificial selection for heat‐tolerant coral reefs (Van Oppen et al. 2015). Such interventions may become increasingly valuable for managers as we face rapid environmental changes from climate change, invasive species, and continued anthropogenic impacts to wildlife populations (Schlaepfer et al. 2005). However, the application of such a technique must weigh several trade‐offs—as any degree of selection will inevitably lower *N*_e_ of the population and perhaps eliminate adaptive genetic variation that is not useful now but may be in the future (i.e., adaptive potential) (Pearse 2016). A precautionary approach suggests preservation of neutral diversity should remain the standard until evidence suggests a better approach is warranted more broadly (Kardos et al. 2021) and for TDC mitigation strategies specifically (Harder et al. 2024). We hope that our work to explore alternative approaches, in a controlled setting with low consequence of failure, is useful to shed light on some of these important questions in species conservation.

## Author Contributions

**Kurt C. Heim:** conceptualization (supporting), data curation (equal), formal analysis (lead), investigation (lead), methodology (lead), project administration (supporting), visualization (lead), writing – original draft (lead), writing – review and editing (lead). **Avril M. Harder:** conceptualization (equal), data curation (equal), formal analysis (supporting), investigation (equal), methodology (equal), writing – review and editing (supporting). **Laurie A. Earley:** conceptualization (supporting), funding acquisition (equal), project administration (equal), writing – review and editing (supporting). **Paul Boynton:** investigation (supporting), project administration (equal), resources (equal), supervision (equal), writing – review and editing (supporting). **Shane Hanlon:** investigation (supporting), project administration (supporting), resources (equal), supervision (equal), writing – review and editing (supporting). **Jacques Rinchard:** data curation (supporting), formal analysis (supporting), investigation (supporting), project administration (supporting), writing – review and editing (supporting). **Kevin Kelsey:** investigation (supporting), project administration (supporting), resources (supporting), writing – review and editing (supporting). **Stacey A. Nerkowski:** project administration (equal), supervision (equal), writing – review and editing (supporting). **Nathan R. Campbell:** methodology (equal), writing – review and editing (supporting). **William R. Ardren:** conceptualization (lead), data curation (supporting), formal analysis (supporting), funding acquisition (equal), investigation (supporting), methodology (supporting), project administration (equal), resources (equal), supervision (equal), writing – review and editing (supporting).

## Funding

This work was supported by the Great Lakes Fishery Commission.

## Conflicts of Interest

Nathan Campbell is the owner of GTseek LLC, which developed the GT‐seq genotyping panel and generated some of the data used in this study. This affiliation is disclosed in the interest of transparency. The author reports no financial interests related to the study outcomes.

## Supporting information


**File S1:** Development and genetic monitoring of a putatively thiamine deficiency complex tolerant Atlantic salmon broodstock.
**Figure S1:** Maternal thiamine vitamer concentration compared to offspring survival. Spearmans Rho, a nonparametric measure of correlation, is shown above each plot.
**Figure S2:** Hazard ratio (relative risk of offspring survival) compared to egg free thiamine in 2016, 2017, and 2018 showing values on original scale (left panels) and log transformed (right panel). The best fitting linear model prediction is shown on right panels.
**Figure S3:** Depictions of steps involved in marking individuals via parentage‐based tagging (PBT). During gamete collection (panel A), a genetic sample is collected from each parent and stored on an annotated piece of chromatography paper (panel B) that assigns unique IDs to each individual, family, and “red bucket.” After fertilization, five family groups are combined in a red bucket (panel C), enumerated, and laid in labeled egg trays within the hatchery (panel D). After eye‐up, egg‐lots are combined to form discrete “PBT groups” with known family groups unique to that lot, that are then transferred to rearing units until stocking as fry or 1.5‐year‐old smolts (panel E).
**Figure S4:** The distribution of SNPs in final GT‐seq marker panel across Atlantic Salmon chromosomes.
**Figure S5:** Trends in total thiamine (panel A), free thiamine (panel B), ratio of free thiamine to total thiamine (panel C), and all vitamers together (TH, free thiamine; THH, total thiamine; TMP, thiamine monophosphate; TPP, thiamine pyrophosphate; panel D).
**Figure S6:** Allele frequency (AF) comparison of broodstock Founders that did and did not produce surviving offspring at TDC‐associated SNPs (panel A, *n* = 34 loci) along with AF comparisons of MAX‐Gen and TDC‐Tolerant offspring stocked in Lake Champlain in 2021 2023 at these same loci. Note each represents a locus. Panel C depicts AF of Founders and Descendants at these TDC‐associated SNPs—separated by results of statistical tests (i.e., AF differ in Founders and Descendants | AF differs in Founders but not Descendants | AF differs in Descendants but not Founders). Panels D and E depict AF differences in Founders (panel D) and descendants (panel E) at 160 neutral loci. Red points indicate statistical significance in AF test (*p* < 0.05).
**Table S1:** Summary of 2444 landlocked Atlantic salmon genotyped at SNPs associated with maturation and sea age derived from studies on Anadromous populations.
**Table S2:** Allele frequency information for putatively TDC‐associated SNPs of Founders that did and did not produce surviving offspring (first set of comparisons) and their descendants which are separated into two experimental broodstock lineages.
**Table S3:** Stocking targets for Atlantic Salmon marked with parentage‐based tagging (PBT) for 2021–2025. Each line represents two uniquely identifiable release groups marked with PBT (i.e., known parentage) that includes one half TDC‐Tolerant and one‐half MAX‐Gen offspring. Therefore, there are 18 PBT groups released annually, inclusive of 357,000 individuals annually (178,500 MAX‐Gen, 178,500 TDC‐Tolerant).

## Data Availability

Data and supporting R scripts presented in this article are publicly available at the U.S. Fish and Wildlife Service data repository ServCat and can be accessed at the following link. ServCat—Generic Dataset – (Code: 190614). https://iris.fws.gov/APPS/ServCat/Reference/Profile/190614.
