# Supplementary material for: Development and Genetic Monitoring of a Putatively Thiamine Deficiency Complex Tolerant Atlantic Salmon Broodstock
Source: Ecol Evol. 2026 Aug 7;16(8):e74122. doi: 10.1002/ece3.74122 (PMC13451162; doi:10.1002/ece3.74122)
Supplement: Supplementary file 1 [file ECE3-16-e74122-s001.pdf]

Supporting Information File 1: Development and genetic monitoring of a putatively thiamine deficiency complex tolerant Atlantic salmon broodstock

Kurt C. Heim[1], Avril M. Harder[2]*, Laurie A. Earley[1], Paul Boynton[3], Shane Hanlon[4], Jacques Rinchard[5], Kevin Kelsey[6], Stacey A. Nerkowski[7], Nathan R. Campbell[8], William R. Ardren[1,9]

[1]U.S. Fish and Wildlife Service, Lake Champlain Fish and Wildlife Conservation Office, Essex Junction, VT 05452, USA

[2]College of Forestry, Wildlife and Environment, Auburn University, Auburn, AL 36849, USA

[3]U.S. Fish and Wildlife Service, White River National Fish Hatchery, Bethel, VT 05032-9130, USA

[4]U.S. Fish and Wildlife Service, Dwight D. Eisenhower National Fish Hatchery, North Chittenden, VT 05032-9130, USA

[5]State University of New York Brockport, Department of Environmental Science and Ecology, Brockport, NY 14420, USA

[6]Vermont Fish and Wildlife Department, Ed Weed Fish Culture Station, Grand Isle, VT, USA

[7]U.S. Fish and Wildlife Service, Northeast Fishery Center, Lamar, PA 16848, USA

[8]GTseek LLC, Twin Falls, ID, USA

[9]U.S. Fish and Wildlife Service, Silvio O. Conte National Fish and Wildlife Refuge Complex, Hadley, MA 01035, USA

*Current address: Genome Sequencing Center, HudsonAlpha Institute for Biotechnology, Huntsville, Alabama 35806, USA

ORCID INFORMATION:
Heim: 0000-0001-7371-4002
Harder: 0000-0001-7371-4002
Rincard: 0000-0002-6247-2551

Corresponding Author: Kurt C. Heim, kurt_heim@fws.gov

METHODS

SNP discovery and ranking for potential inclusion in GT-seq panel

We developed a genotyping-in-thousands (GT-seq) marker panel (Campbell et al. 2015) for the primary purpose of genetic parentage analysis to facilitate parentage-based tagging of Atlantic salmon (*Salmo salar*)(Steele et al. 2019). Secondary intended purposes were to determine genetic sex, provide a set of markers associated with thiamine deficiency complex (TDC) tolerance, and examine markers known to be associated with fitness related traits in sea-run Atlantic salmon. The following section describes how we identified and selected candidate SNPs for potential inclusion in this panel.

We selected 95 individuals that were founding parents of our two captive experimental broodstocks (2016 = 31, 2017 = 32, 2018 = 32) to use as ascertainment samples for restriction-site associated DNA sequencing (RAD-seq) and single nucleotide polymorphism (SNP) discovery. The samples included individuals that either did (n = 53) or did not (n = 42) produce surviving offspring within survival trials conducted in 2016, 2017, and 2018. At Abernathy Fish Technology Center, genomic DNA was extracted from each tissue sample using a DNeasy Blood and Tissue Kit (Qiagen, Inc. Valencia, CA) according to the manufacturer's protocol and sent to Floragenex Inc. where one SbfI restriction site-associated DNA sequencing (RADseq) library was prepared and sequenced on a Illumina HiSeq Lane.

We received RADseq data from Floragenex, Inc., in FASTQ file format. Sequences were demultiplexed using process_radtags in STACKS v2.64 (Catchen et al. 2013). Demultiplexed sequences were aligned to the Atlantic Salmon reference genome (S. salar ICSASG_v2 assembly, NCBI accession GCA_000233375.4 (Lien et al. 2016) using the BWA-MEM algorithm of the Burrows-Wheeler Aligner (BWA) (Li and Durbin 2009; Li 2013). We used SAMtools (Li et al. 2009; Danecek et al. 2021) to sort, filter for proper pairs, and index paired end binary alignment map (BAM) files. Variants were called using BCFtools mpileup and call commands (Li 2011), and a variable call format file (VCF (Danecek et al. 2011) was produced using BCFtools. We first identified and prioritized ~500 SNPs spread across chromosomes with minor allele frequencies between 0.2-0.4 that were included in the candidate list for the final panel. These SNPs are intended for primary use in parentage analysis.

Next, we sought to identify a suite of TDC-tolerance associated SNPs for potential inclusion in the final panel. We conducted association tests implemented in ANGSD (Sand Korneliussen et al. 2014) to test for differences in allele frequencies between samples collected from parents with surviving offspring and no surviving offspring. We conducted the test using the implementation of the likelihood ratio test described in (Kim et al. 2011)with a minimum mapping quality score (minMapQ) of 20, a minimum base quality score (minQ) of 20, and the SAMtools genotype likelihood model, GL 1 (Li et al. 2009). All analyses implemented in ANGSD included only those sites that were represented in 50% of the samples (minInd). We conducted two association tests, one with both sexes and one with only females and developed a ranked-list of SNPs based on the likelihood ratio statistic (LRT) from each association test. All subsequent prioritization steps selected SNPs from the association test with LRT >= 10 in at least one of the association tests.

We prioritized TDC-associated SNPs for potential inclusion in the GT-seq panel by (1) selecting the top-25 SNPs from each association test including all individuals (n = 25 SNPs) and female only tests (n=25 NPS) (2) identifying SNPs that were in or near differentially expressed genes identified in Harder et al. (2020) (3) identifying SNPs resulting in a nonsynonymous change in an annotated gene and (4) identifying SNPs located in an intron of an annotated gene or resulting in a synonymous change in an exon (i.e., potential regulatory consequences). In sum we selected 146 putatively TDC-associated SNPs as candidate loci for inclusion in the GT-seq panel.

We also searched published literature for SNPs of potential interest for inclusion in the GT-seq panel including SNPs associated with sea age and maturation. These included seven SNPs published in Aykanat et al. (2016) including the vestigial-like family member 3 gene (vgll3) that explains 39% of phenotypic variation in anadromous salmon (Barson et al. 2015), nine published in (Boulding et al. 2019) and five in (Waters et al. 2020).

GT-seq marker panel development

The VCF file along with chromosome position of candidate SNPs were sent to GTSEEK (https://gtseek.com/) for Genotyping-in-Thousands by Sequencing (GT-Seq; Campbell et al. 2015) panel design. The panel design process involves iterative testing of different sets of SNPs (i.e., library prep, sequencing, genotyping) for overall performance to achieve a final set of SNPs that produce reliable genotypes. In brief, GT-seq primers were designed using GTseek's proprietary primer design pipeline, which screens candidate primers for thermodynamic stability, genomic specificity, and multiplex compatibility. Primer candidates were required to have melting temperatures of 58–63°C, lengths of 17–25 bp, and 20–80% GC content. Secondary structure filters were applied to remove primers exhibiting predicted hairpin Tm values >50°C. To minimize undesired cross-reactivity within the multiplex, each candidate primer was evaluated for 3′ heterodimer formation against all previously accepted primers, using the 3′-most 10 bases of both sequences. Because accepted primers already include their Illumina overhang sequences, heterodimer screening accounts for interactions involving both the target-specific region and the attached Illumina tag. Any candidate exhibiting a predicted 3′ heterodimer melting temperature of ≥12°C with any previously accepted primer was excluded. Primers passing all criteria were appended with standard Illumina tags and assembled into draft multiplex pools.

Draft multiplex primers were synthesized and tested using the standard two-PCR GT-seq workflow modified by using the Nate's Plates GT-seq tagging and normalization kits for PCR2 (GTseek, Twin Falls, ID). All test libraries were sequenced on an Illumina platform. Amplicon performance was evaluated based on target-capture efficiency, read-depth uniformity, allelic balance, and absence of secondary or off-target products. Loci exhibiting low efficiency, excessive background, or unstable allele profiles were omitted, and revised panels were iteratively re-tested. Optimization continued until the multiplex achieved ≥50% target-capture efficiency with consistent amplification across samples, at which point a finalized primer pool was prepared for production genotyping.

Genotyping at the GT-seq marker panel for initial testing and PBT validation

With a final panel developed, we genotyped a set of 2,444 samples to perform initial testing of the panel for parentage analysis, validate our PBT approach, and examine the association of TDC-associated SNPs with offspring survival. These 2,444 samples included all hatchery

broodstock from MAX-Gen and TDC-Tolerant that were spawned in 2019, 2020, and 2021 (n = 1930 broodstock samples) collected during Fall spawning. Additionally, we collected 419 PBT validation samples from PBT-marked fish immediately prior to stocking. Lastly, all 95 ascertainment samples (i.e., that RAD-seq was performed on, and whose offspring participated in survival trials) were genotyped.

Genomic DNA was extracted from each tissue sample using a DNeasy Blood and Tissue Kit (Qiagen, Inc. Valencia, CA) according to the manufacturer's protocol. Initial extractions were concentrated by drying down 35µL of each (room temperature for approximately five days) and rehydrating in 10 µL of molecular grade water. The GT-Seq protocol included pre-amplification of a primer pool of 208 loci and 3 sdy sex probes, and barcode amplification resulting in a unique barcode for each sample. This was followed by a normalization step using a SequalPrep Normalization Plate Kit (Thermo Fisher Scientific, Waltham, MA) and pooling the normalized product for amplicon size selection using AMPure XP beads (Beckman Coulter Brea, CA) and a magnetic rack. Quantification of libraries was done by qPCR using KAPA Library Quantification Kit for Illumina platforms (Roche Basel, Switzerland). Libraries were then normalized to 4nM and verified using a Quibit Fluorometer (Invitrogen Life Technologies Waltham, MA) according to the manufacturer's protocol, and were processed on a NextSeq 1000 (Illumina, Inc.).

To determine laboratory error rate, a quality assessment/quality control (QA/QC) was performed by selecting one column (eight samples) from each 96-well plate of extracted DNA, and building a new library based on those samples. The GT-Seq protocol was as described above, except that the QA/QC library was processed on a MiSeq (Illumina, Inc.). Genotype data were extracted from the resulting sequence data using the bioinformatic scripts developed by Campbell et al. (2015).

Implementation of Parentage-based tagging

After developing the GT-seq marker panel and curating two broodstocks (TDC-tolerant, MAX-gen) we implemented PBT to genetically tag fish according to a series of experimental release groups. Spawning broodstock was done at White River National Fish Hatchery. We created single-parent crosses, and collected a genetic sample from each individual. Tissue samples were stored on pre-labeled chromatography paper, where each individual and family was given a unique ID. After fertilization within discrete bowls, each unique family is given two minutes for fertilization to occur before five families are combined into a uniquely labeled red BUCKET (**Figure S4**, Main Text). The data sheets also assign BUCKET IDs to each set of five families. Each labeled BUCKET is transferred to inside the facility, and inventoried (i.e., eggs counted) into unique heath trays for incubation. During incubation, a parent database is developed linking each individual to a family, each family to a BUCKET, and each BUCKET to an incubation tray. Therefore, after the completion of spawning, we know exactly which pairs were created and which pairs were uniquely present within heath trays.

Following eye-up of eggs, each tray is re-inventoried to determine the viable egg count within each tray. Next, a tray-assignment list is developed to combine eggs into groups of 25-30 families (i.e., PBT groups). Tray allocation seeks to (1) achieve a target total egg-number for each PBT group according to various stocking targets (2) consistent number of families per PBT group (3) normalize the contribution per BUCKET to each PBT group (4) have equal

representation across PBT groups in terms of BY contributions (e.g., first or second year spawners) and (5) represent families produced across the range of spawning dates.

Hatchery staff combine contents of health trays according to the tray-assignment database, and distribute the combined lots of eggs (now labeled with a PBT group ID) to Adirondack Hatchery (ADK) in NY and Dwight D. Eisenhower National Fish hatchery (DDE) in Vermont. The PBT groups at ADK are incubated over winter and stocked in NY tributaries to Lake Champlain as un-fed fry in the spring, whereas PBT groups sent to DDE are raised to smolt size (1.5 years, ~160 mm) in separate rearing units and released in two calendar years after their fertilization (e.g., BY20 released as smolts in Spring 2022). At the time of release, counts of stocked fish are estimated.

Ultimately, we will use genetic assignment of resampled fish (i.e., post-release, within the Lake or Rivers) to match individuals to (1) a family (2) a bucket and (3) a PBT group. It is worth noting that this provides three levels at which we will be able to statistically analyze the data; recaptures can be assessed at the family, bucket, or PBT group level. Because the recapture rate depends on the number released, it is worth discussing how well we can estimate the denominator for each of these three levels. Because we have counts of fish stocked that occur at the PBT group level, this is the level with the most accurate denominator (i.e., recaptures per PBT group/number released per PBT group). Next, because we know the total number of eggs contributed by each BUCKET to a PBT group, we can also estimate BUCKET level contributions by making some assumptions. If fish within each BUCKET had equal survival within the hatchery rearing period, then an appropriate denominator for BUCKET i is the total number of fish released multiplied by the fraction of eggs contributed by BUCKET i relative to the total number of eggs contributing to the PBT group. This would allow us to estimate returns per BUCKET with a scaling factor for expected number of fish released per bucket. A similar approach could be developed, with certain assumptions, to develop a family-level scaling factor so that recaptures can be analyzed per family. In any of these possible scenarios, the assumptions of each approach need further consideration and analysis.

We developed 18-PBT groups in coordination with members of the Lake Champlain Fisheries Technical committee to simultaneously address a suite of applied research questions of interest to State agencies (**Table S3**). First, we are evaluating the overall performance of MAX and TDC broodstocks – all stocking events include an equal number of fish from the two broodstocks (9 PBT groups are MAX, each with an associated TDC-tolerant group = 18 total). We are also evaluating (1) the efficacy of fry-stocking (4 river-systems) vs. smolt stocking (3 river systems) (2) hard release vs. soft release (i.e., net-pen acclimation) in the Saranac River, NY (Connerton et al. 2022), and imprinting vs. survival tradeoffs related to smolt stocking location in the Winooski River, VT with a paired radio-telemetry study (Heim et al. 2025). Finally, all of these release groups provide opportunities to evaluate the relative reproductive success of each broodstock via widespread genetic sampling of adults, subadults, and offspring and use of mutli-generational pedigree analysis. The program identified a five-year release period as a target for this study (i.e., 18 groups, released from 2020 – 2024).

Additional allele frequency difference testing

One of our stated aims in developing the TDC-Tolerant broodstock was to increase potentially adaptive alleles; therefore, we performed a simple exploratory analysis to characterize allele frequency differences in two sets of samples (1) broodstock founders that did and did not produce surviving offspring in survival trials and (2) hatchery fish stocked in Lake Champlain from the two resulting broodstock lineages, MAX-Gen and TDC-Tolerant. The first set of comparisons included feral salmon returning to Ed Weed Fish Culture Station used in survival trials that produced surviving offspring (n = 40) and did not produce surviving offspring (n = 55) in the absence of thiamine treatment. Chi-squared tests were implemented to test allele frequency differences between founders with and without surviving offspring ($\alpha = 0.05$). This was done for the set of putatively TDC-associated SNPs (n = 35) as well as the set of polymorphic neutral SNPs included in our final panel (n = 164). We do not make corrections for multiple hypothesis testing (e.g., Bonferroni correction) but rather report the expected number of Type I errors expected (based on the number of tests performed) and compare this to the number of observed significant tests (Waples 2015). These tests generally represent the question 'how different are broodstock founders' at the TDC-associated SNPs (with an expectation for strong differences, as these individuals are the same used in identifying the TDC-associated SNPs in panel development) and at neutral loci (no expectation for differences).

The next set of tests, on hatchery released fish from MAX-Gen and TDC-Tolerant lineages, represent the general question 'how different are the resulting lineages" after several generations of captive breeding and lineage separation (**Table 1, Main Text**). These samples are from hatchery fish (smolts and fry) that were released into Lake Champlain for ongoing assessment in the wild and are descendants of the 2016-2018 founding individuals. These samples included fish released from 2021 - 2023 (MAX-Gen = 310, TDC-Tolerant = 369) and also used in the aforementioned PBT validation analysis. With these samples, we used the same chi-squared testing procedure described above with the TDC-associated SNPs and neutral SNPs.

RESULTS

Additional allele frequency difference testing

Considering the 'Founder' genotypes (n = 95, i.e., individuals from survival trials used as founders of our two broodstocks), allele frequencies differed between those with and without surviving offspring at 11/34 (32%) of the TDC-associated SNPs (**Figure S6**, **Table S2**) when ~two would be expected by chance alone (i.e., 0.05 x 34 tests). Considering samples of hatchery fish stocked in Lake Champlain (i.e., 'Descendants') allele frequencies differed between MAX-Gen and TDC-Tolerant lineages at 13/34 (38%) of these SNPs; however, only five of these SNPs were consistently significant in Founders and Descendants (**Figure S6C**). In these five SNPs, the allele associated with higher survival among Founders, was also represented at a higher frequency in the TDC-Tolerant Descendants (relative to MAX-Gen Descendants). At six loci, comparisons were significant in Founders but not so in Descendants. Here, the allele associated with survival from Founders occurred at a higher (but not significantly higher) frequency in TDC-Tolerant Descendants at 4/6 SNPs. At eight loci, comparisons were significant in Descendants but not in Founders, and in 6/8 these loci the allele associated with higher Founder offspring survival was represented at a higher frequency (but not significantly higher) in TDC-Tolerant Descendants. At 15 loci, allele frequency comparisons were not significant in either the Founders or Descendants.

Considering the neutral SNPs, 8/160 (5%) of loci had significant allele frequency differences between Founders with and without surviving offspring, compared to eight (0.05 * 160) that would be expected due to chance. Considering Descendants at the neutral SNPs, 55 loci (34%) were significantly different between MAX-Gen and TDC-Tolerant samples, again with only eight expected to occur owing to chance.

References:

Barson, N. J., T. Aykanat, K. Hindar, M. Baranski, G. H. Bolstad, P. Fiske, C. Jacq, A. J. Jensen, S. E. Johnston, S. Karlsson, M. Kent, T. Moen, E. Niemelä, T. Nome, T. F. Næsje, P. Orell, A. Romakkaniemi, H. Sægrov, K. Urdal, J. Erkinaro, S. Lien, and C. R. Primmer. 2015. Sex-dependent dominance at a single locus maintains variation in age at maturity in salmon. Nature 528(7582):405–408.

Boulding, E. G., K. P. Ang, J. A. K. Elliott, F. Powell, and L. R. Schaeffer. 2019. Differences in genetic architecture between continents at a major locus previously associated with sea age at sexual maturity in European Atlantic salmon. Aquaculture 500(April 2018):670–678.

Campbell, N. R., S. A. Harmon, and S. R. Narum. 2015. Genotyping-in-Thousands by sequencing (GT-seq): A cost effective SNP genotyping method based on custom amplicon sequencing. Molecular Ecology Resources 15(4):855–867.

Catchen, J., P. A. Hohenlohe, S. Bassham, A. Amores, and W. A. Cresko. 2013. Stacks: An analysis tool set for population genomics. Molecular Ecology 22(11):3124–3140.

Connerton, M. J., J. R. Lantry, C. R. Bronte, and S. R. Lapan. 2022. Origin, Postrelease Survival, and Imprinting of Pen-Acclimated and Direct-Stocked Chinook Salmon in Lake Ontario. North American Journal of Fisheries Management 42(3):713–740.

Danecek, P., A. Auton, G. Abecasis, C. A. Albers, E. Banks, M. A. DePristo, R. E. Handsaker, G. Lunter, G. T. Marth, S. T. Sherry, G. McVean, and R. Durbin. 2011. The variant call format and VCFtools. Bioinformatics 27(15):2156–2158.

Danecek, P., J. K. Bonfield, J. Liddle, J. Marshall, V. Ohan, M. O. Pollard, A. Whitwham, T. Keane, S. A. McCarthy, and R. M. Davies. 2021. Twelve years of SAMtools and BCFtools. GigaScience 10(2).

Heim, K. C., W. R. Ardren, J. L. Withers, Z. J. Eisenhauer, M. A. Mensinger, and T. Castro-Santos. 2025. Spatial variation in landlocked Atlantic Salmon smolt survival associated with dam passage, avian predation, and stocking location. North American Journal of Fisheries Management.

Kim, S. Y., K. E. Lohmueller, A. Albrechtsen, Y. Li, T. Korneliussen, G. Tian, N. Grarup, T. Jiang, G. Andersen, D. Witte, T. Jorgensen, T. Hansen, O. Pedersen, J. Wang, and R. Nielsen. 2011. Estimation of allele frequency and association mapping using next-generation sequencing data. BMC Bioinformatics 12.

Li, H. 2013. Aligning sequence reads, clone sequences and assembly contigs with BWA-MEM.

Li, H., and R. Durbin. 2009. Fast and accurate short read alignment with Burrows-Wheeler transform. Bioinformatics 25(14):1754–1760.

Li, H., B. Handsaker, A. Wysoker, T. Fennell, J. Ruan, N. Homer, G. Marth, G. Abecasis, and R. Durbin. 2009. The Sequence Alignment/Map format and SAMtools. Bioinformatics 25(16):2078–2079.

Lien, S., B. F. Koop, S. R. Sandve, J. R. Miller, M. P. Kent, T. Nome, T. R. Hvidsten, J. S. Leong, D. R. Minkley, A. Zimin, F. Grammes, H. Grove, A. Gjuvsland, B. Walenz, R. A.

Hermansen, K. Von Schalburg, E. B. Rondeau, A. Di Genova, J. K. A. Samy, J. Olav Vik, M. D. Vigeland, L. Caler, U. Grimholt, S. Jentoft, D. Inge Våge, P. De Jong, T. Moen, M. Baranski, Y. Palti, D. R. Smith, J. A. Yorke, A. J. Nederbragt, A. Tooming-Klunderud, K. S. Jakobsen, X. Jiang, D. Fan, Y. Hu, D. A. Liberles, R. Vidal, P. Iturra, S. J. M. Jones, I. Jonassen, A. Maass, S. W. Omholt, and W. S. Davidson. 2016. The Atlantic salmon genome provides insights into rediploidization. Nature 533:200–205.

Sand Korneliussen, T., A. Albrechtsen, and R. Nielsen. 2014. ANGSD: Analysis of Next Generation Sequencing Data.

Steele, C. A., M. Hess, S. Narum, and M. Campbell. 2019. Parentage-Based Tagging: Reviewing the Implementation of a New Tool for an Old Problem. Fisheries 44(9):412–422.

Waters, S., G. Sel, M. S. Waters, J. Ødegård, S. A. Korsvoll, T. Moen, S. Lien, C. R. Primmer, and N. J. Barson. 2020. Beyond large effect loci : large scale GWAS reveals a mixed large effect and polygenic architecture for age at maturity of Atlantic salmon. Genetics Selection Evolution:1–11.

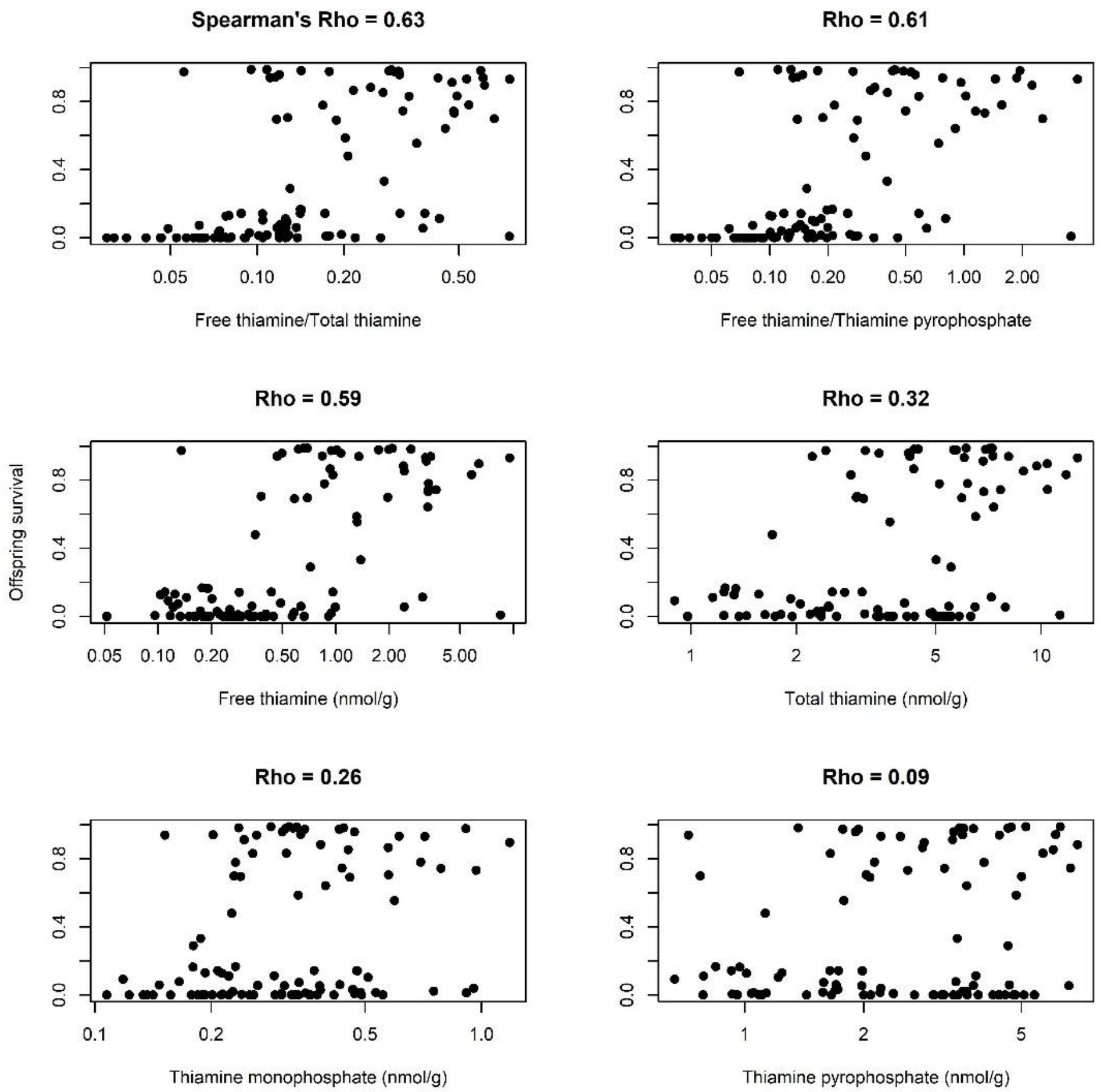

Figure S1. Maternal thiamine vitamer concentration compared to offspring survival. Spearmans Rho, a nonparametric measure of correlation, is shown above each plot.

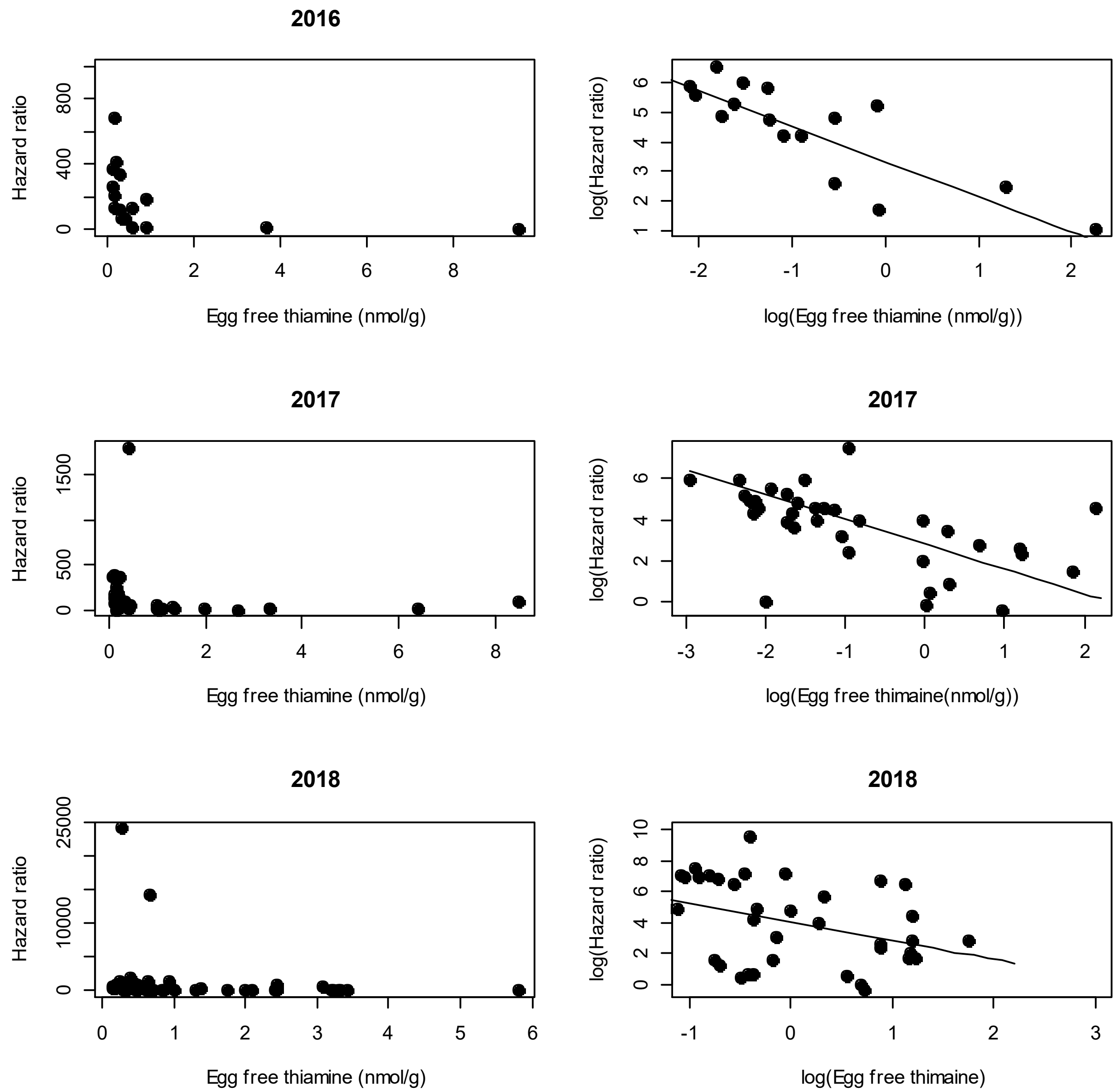

Figure S2. Hazard ratio (relative risk of offspring survival) compared to egg free thiamine in 2016, 2017, and 2018 showing values on original scale (left panels) and log transformed (right panel). The best fitting linear model prediction is shown on right panels.

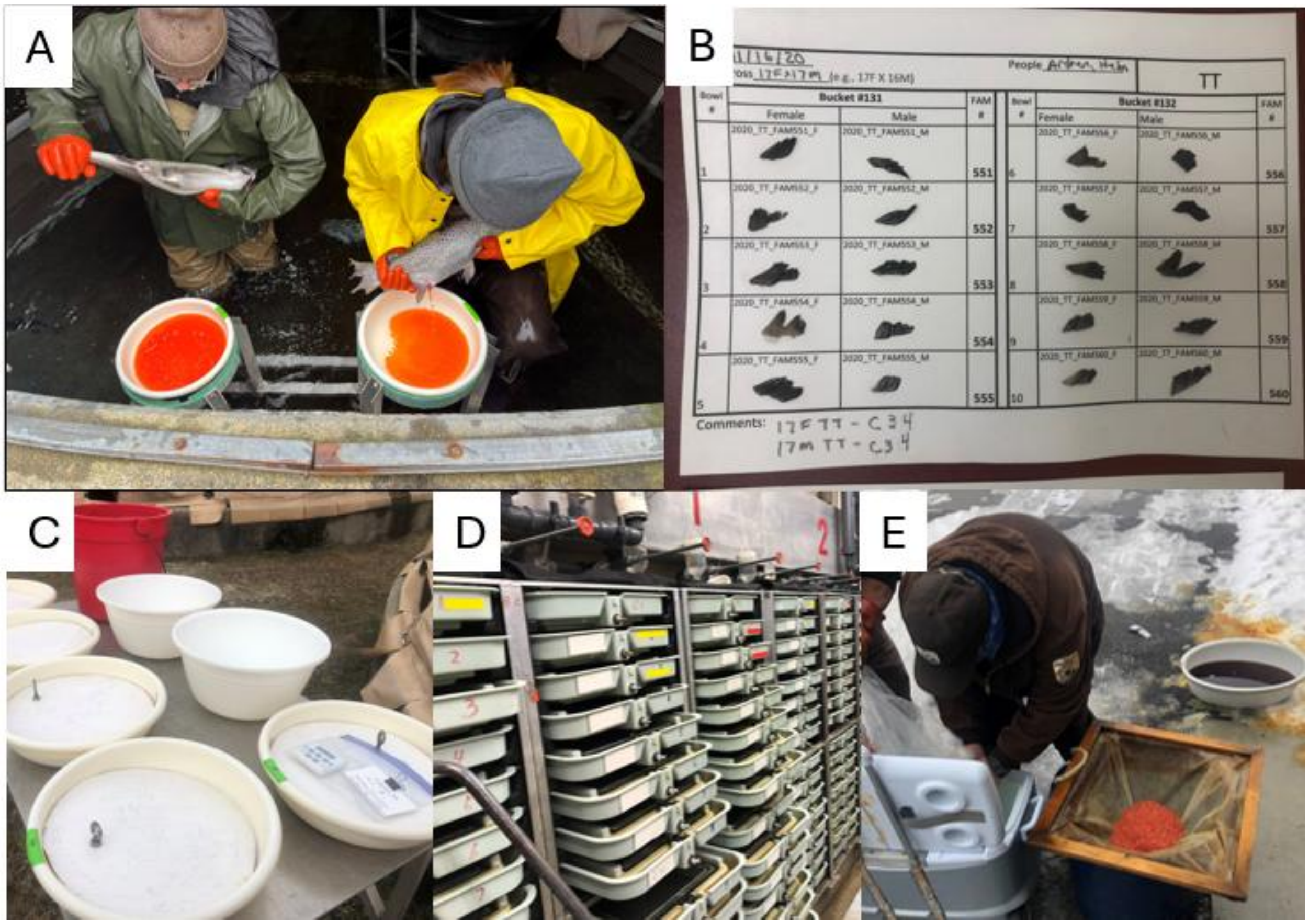

Figure S3. Depictions of steps involved in marking individuals via parentage-based tagging (PBT). During gamete collection (panel **A**), a genetic sample is collected from each parent and stored on an annotated piece of chromatography paper (panel **B**) that assigns unique IDs to each individual, family, and 'red bucket'. After fertilization, five family groups are combined in a red bucket (panel **C**), enumerated, and laid in labeled egg trays within the hatchery (panel **D**). After eye-up, egg-lots are combined to form discrete 'PBT groups" with known family groups unique to that lot, that are then transferred to rearing units until stocking as fry or 1.5-year-old smolts (panel **E**).

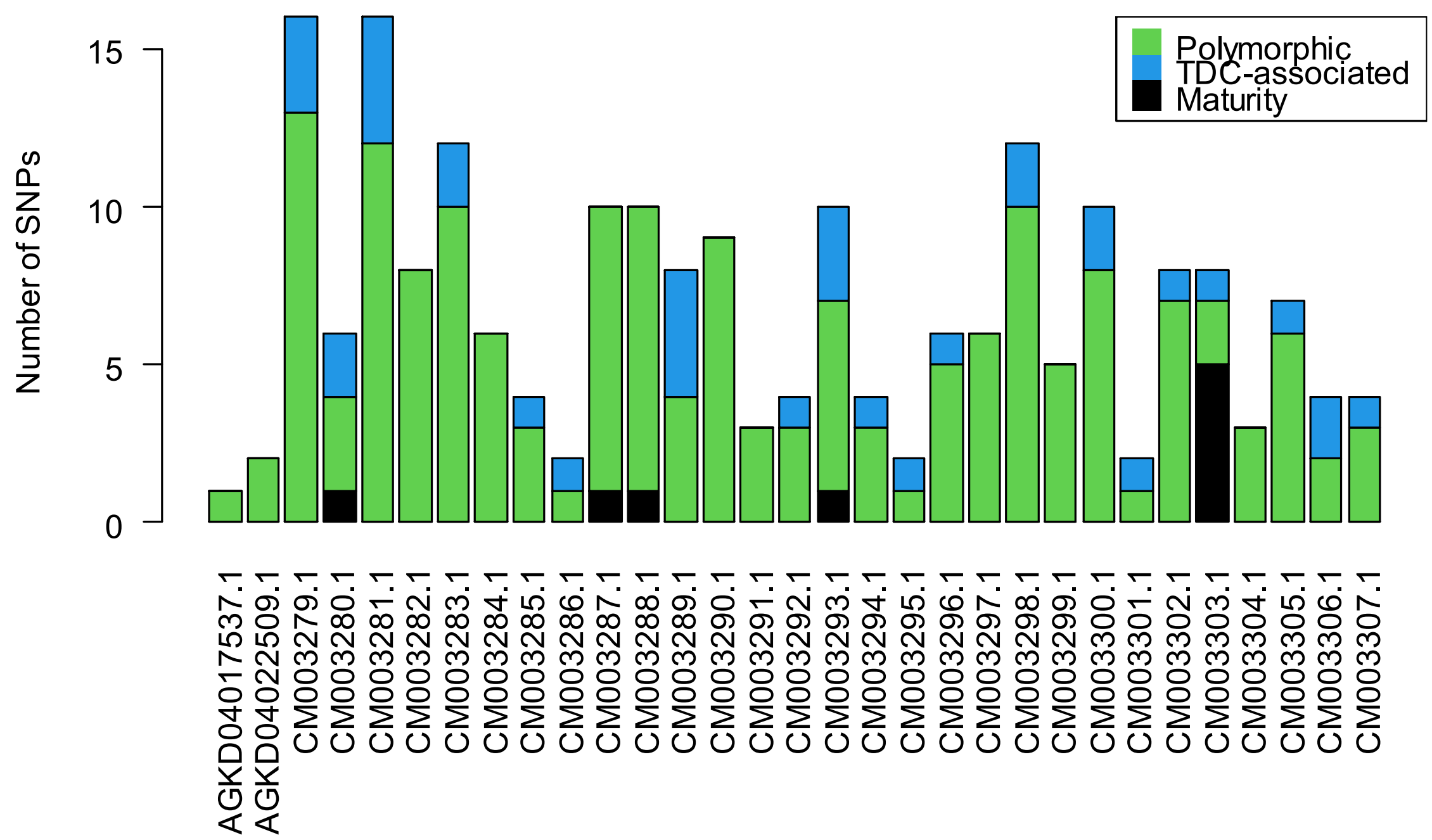

Figure S4. The distribution of SNPs in final GT-seq marker panel across Atlantic Salmon chromosomes.

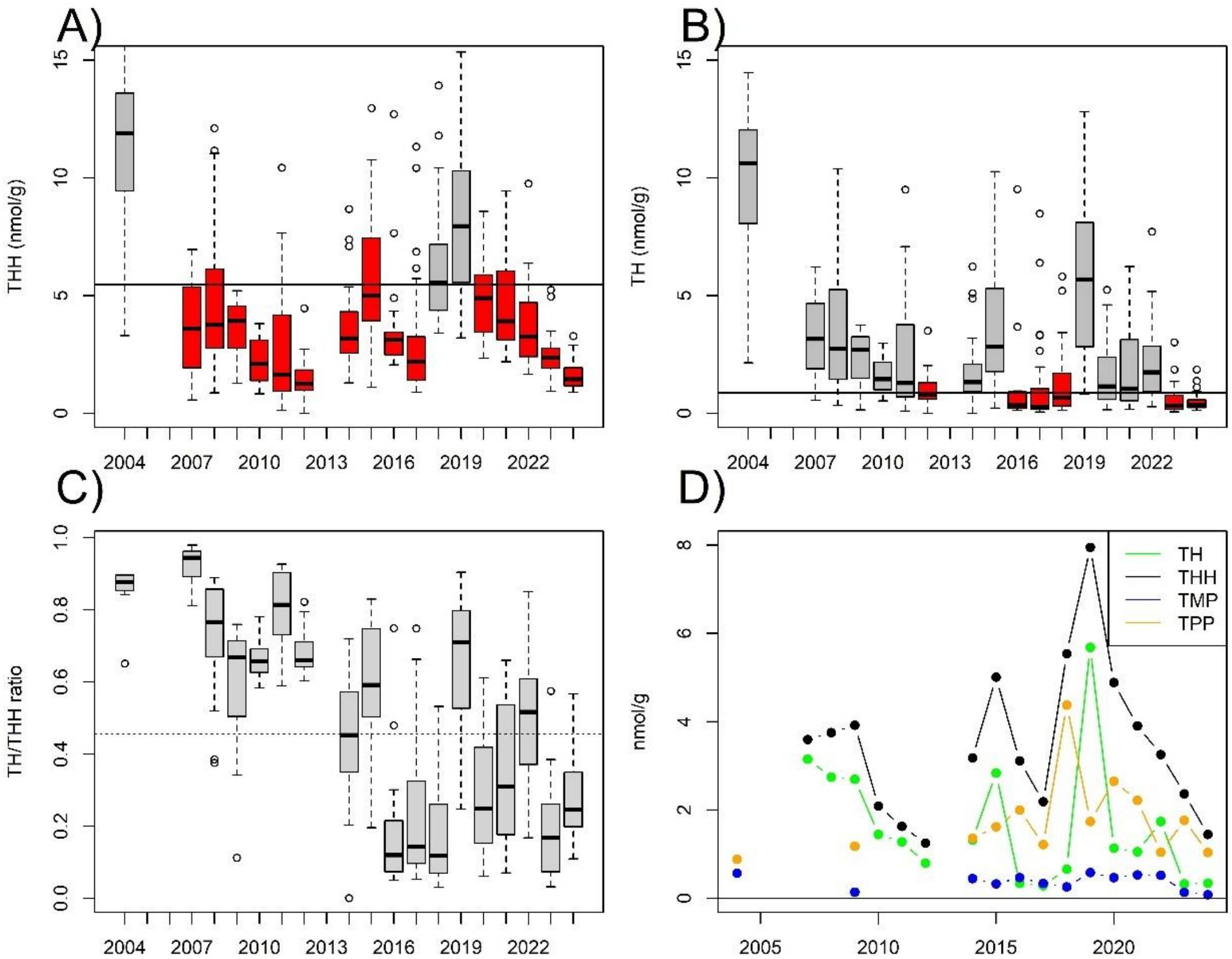

Figure S5. Trends in total thiamine (panel A), free thiamine (panel B), ratio of free thiamine to total thiamine (panel C), and all vitamers together (TH = free thiamine, THH = total thiamine, TMP = thiamine monophosphate, TPP = thiamine pyrophosphate, panel D).

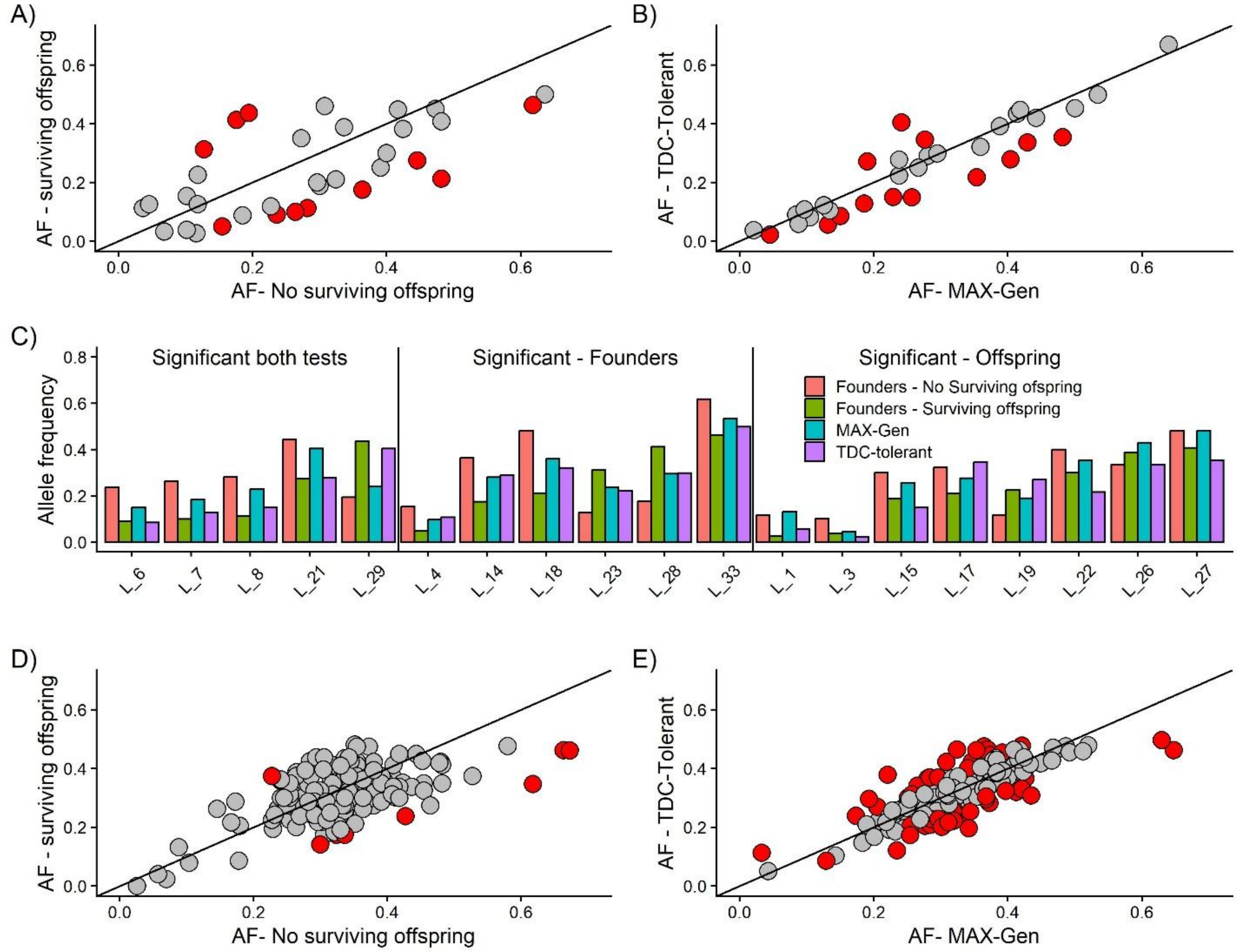

Figure S6. Allele frequency (AF) comparison of broodstock Founders that did and did not produce surviving offspring at TDC-associated SNPs (panel **A,** n = 34 loci) along with AF comparisons of MAX-Gen and TDC-Tolerant offspring stocked in Lake Champlain in 2021-2023 at these same loci. Note each represents a locus. Panel **C** depicts AF of Founders and Descendants at these TDC-associated SNPs – separated by results of statistical tests (i.e., AF differ in Founders and Descendants | AF differs in Founders but not Descendants | AF differs in Descendants but not Founders). Panels **D** and E depict AF differences in Founders (panel **D**) and descendants (panel **E**) at 160 neutral loci. Red points indicate statistical significance in AF test ($p < 0.05$).

Table S1. Summary of 2,444 landlocked Atlantic salmon genotyped at SNPs associated with maturation and sea age derived from studies on Anadromous populations.

Position	Name	Trait	MA	MAF	Miss%	Reference
CM003280.1_26594069	AX-86961236	grilse	T	0.158	0.049	Boulding et al. (2019)
CM003287.1_24904601	chr09_peak	Sea Age	T	0.025	0.018	Barson et al. (2015)
CM003288.1_63130161	AX-96190371	grilse	G	0.151	0.018	Boulding et al. (2019)
CM003293.1_6399839	NADH*	Maturation	Fixed[C]	1.000	0.016	Sinclair-Waters et al. (2020)
CM003303.1_28658151	vgll3 missense 1	Sea Age	Fixed[G]	1.000	0.020	Barson et al. (2015)
CM003303.1_28666148	chr25_peak	Sea Age	Fixed[T]	1.000	0.095	Barson et al. (2015)
CM003303.1_28666898	chr25_peak	Sea Age	Fixed[T]	1.000	0.019	Barson et al. (2015)
CM003303.1_28720779	AKAP11 missense	Sea Age	Fixed[G]	1.000	0.018	Barson et al. (2015)
CM003303.1_28910202	vgll3	Maturation	T	0.025	0.018	Sinclair-Waters et al. (2020)

***Full listed name in original arcticle:** NADH:ubiquinone oxidoreductase subunit S4

Table S2. Allele frequency information for putatively TDC-associated SNPs of Founders that did and did not produce surviving offspring (first set of comparisons) and their descendants which are separated into two experimental broodstock lineages.

		Founder AF comparsions			Descendats AF comparsions			
locus.allele	Abrev.	Surv.	No Surv.	p	MAX-Gen	TDC-tolerant	p	Surv Association
CM003279_1_98569962.A	L_1	0.03	0.12	0.066	0.13	0.06	**0.000**	Lower|Lower
CM003302_1_23671607.A	L_2	0.03	0.07	0.552	0.02	0.04	0.157	Lower|Higher
CM003281_1_24263921.C	L_3	0.04	0.10	0.182	0.05	0.02	**0.030**	Lower|Lower
CM003306_1_13850548.T	L_4	0.05	0.15	**0.042**	0.10	0.11	0.569	Lower|Higher
CM003298_1_47526698.C	L_5	0.09	0.19	0.093	0.09	0.06	0.055	Lower|Lower
CM003289_1_22120190.A	L_6	0.09	0.24	**0.017**	0.15	0.08	**0.000**	Lower|Lower
CM003289_1_20085200.G	L_7	0.10	0.26	**0.009**	0.19	0.13	**0.005**	Lower|Lower
CM003280_1_29470046.T	L_8	0.11	0.28	**0.008**	0.23	0.15	**0.000**	Lower|Lower
CM003281_1_77831154.A	L_9	0.11	0.04	0.078	0.09	0.10	0.884	Higher|Higher
CM003298_1_55869301.T	L_10	0.12	0.23	0.090	0.13	0.10	0.119	Lower|Lower
CM003283_1_62295452.T	L_11	0.13	0.05	0.083	0.10	0.08	0.159	Higher|Lower
CM003300_1_29334100.C	L_12	0.13	0.12	1.000	0.13	0.12	0.927	Higher|Lower
CM003295_1_44918064.T	L_13	0.15	0.10	0.402	0.08	0.09	0.760	Higher|Higher
CM003279_1_84570274.C	L_14	0.18	0.36	**0.007**	0.28	0.29	0.754	Lower|Higher
CM003283_1_60571204.A	L_15	0.19	0.30	0.111	0.26	0.15	**0.000**	Lower|Lower
CM003306_1_13034119.A	L_16	0.20	0.30	0.184	0.24	0.28	0.112	Lower|Higher
CM003305_1_28099823.G	L_17	0.21	0.32	0.127	0.28	0.35	**0.008**	Lower|Higher
CM003280_1_23156675.C	L_18	0.21	0.48	**0.000**	0.36	0.32	0.153	Lower|Lower
CM003293_1_82068928.G	L_19	0.23	0.12	0.077	0.19	0.27	**0.001**	Higher|Higher
CM003286_1_5923963.T	L_20	0.25	0.39	0.060	0.27	0.25	0.553	Lower|Lower
CM003289_1_10836456.C	L_21	0.28	0.45	**0.025**	0.40	0.28	**0.000**	Lower|Lower
CM003294_1_7458099.T	L_22	0.30	0.40	0.205	0.35	0.22	**0.000**	Lower|Lower
CM003279_1_98525186.T	L_23	0.31	0.13	**0.003**	0.24	0.22	0.561	Higher|Lower
CM003285_1_2732679.A	L_24	0.35	0.27	0.326	0.39	0.39	0.937	Higher|Higher
CM003303_1_26726451.C	L_25	0.38	0.42	0.668	0.44	0.42	0.440	Lower|Lower
CM003292_1_59607641.A	L_26	0.39	0.34	0.567	0.43	0.34	**0.001**	Higher|Lower
CM003293_1_30058922.A	L_27	0.41	0.48	0.398	0.48	0.35	**0.000**	Lower|Lower
CM003296_1_12078042.G	L_28	0.41	0.18	**0.001**	0.30	0.30	0.933	Higher|Higher
CM003300_1_2936257.T	L_29	0.44	0.19	**0.001**	0.24	0.40	**0.000**	Higher|Higher
CM003281_1_75171172.G	L_30	0.45	0.42	0.793	0.41	0.43	0.543	Higher|Higher
CM003289_1_15355115.C	L_31	0.45	0.47	0.871	0.42	0.45	0.321	Lower|Higher
CM003307_1_13973510.A	L_32	0.46	0.31	0.056	0.50	0.45	0.099	Higher|Lower
CM003301_1_15353431.G	L_33	0.46	0.62	**0.047**	0.53	0.50	0.212	Lower|Lower
CM003281_1_64867685.C	L_34	0.50	0.64	0.083	0.64	0.67	0.282	Lower|Higher

Table S3. Stocking targets for Atlantic Salmon marked with parentage-based tagging (PBT) for 2021 - 2025. Each line represents two uniquely identifiable release groups marked with PBT (i.e., known parentage) that includes one half TDC-Tolerant and one-half MAX-Gen offspring. Therefore, there are 18 PBT groups released annually, inclusive of 357,000 individuals annually (178,500 MAX-Gen, 178,500 TDC-Tolerant).

State	River	Age	Location	N target
NY	Boquet	Fry	Tributaries	50000
NY	Boquet	Fry	Mainstem	50000
NY	Boquet	Smolt	Various	51500
NY	Ausable	Fry	Various	40000
NY	Saranac	Smolt	Net-pen (soft release)	25750
NY	Saranac	Smolt	River (hard release)	25750
NY	Saranac	Fry	Various	70000
VT	Winooski	Smolt	29 km upstream	22000
VT	Winooski	Smolt	16 km upstream	22000
